# Genomic Epidemiology of the SARS-CoV-2 Epidemic in Cyprus from November 2020 to October 2021: The Passage of Waves of Alpha and Delta Variants of Concern

**DOI:** 10.3390/v15010108

**Published:** 2022-12-30

**Authors:** Andreas C. Chrysostomou, Bram Vrancken, Christos Haralambous, Maria Alexandrou, Antonia Aristokleous, Christina Christodoulou, Ioanna Gregoriou, Marios Ioannides, Olga Kalakouta, Christos Karagiannis, George Koumbaris, Charalambos Loizides, Michail Mendris, Panagiotis Papastergiou, Philippos C. Patsalis, Despo Pieridou, Jan Richter, Markus Schmitt, Christos Shammas, Dora C. Stylianou, Giorgos Themistokleous, the COMESSAR Network, Philippe Lemey, Leondios G. Kostrikis

**Affiliations:** 1 Department of Biological Sciences, University of Cyprus, Aglantzia, Nicosia 2109, Cyprus; 2 Department of Microbiology, Immunology and Transplantation, Rega Institute, KU Leuven, 3000 Leuven, Belgium; 3 Spatial Epidemiology Lab (SpELL), Université Libre de Bruxelles, 1050 Bruxelles, Belgium; 4 Unit for Surveillance and Control of Communicable Diseases, Ministry of Health, Nicosia 1148, Cyprus; 5 Microbiology Department, Larnaca General Hospital, Larnaca 6301, Cyprus; 6 Department of Molecular Virology, Cyprus Institute of Neurology and Genetics, Nicosia 2371, Cyprus; 7 NIPD Genetics, Nicosia 2409, Cyprus; 8 Microbiology Department, Nicosia General Hospital, Nicosia 2029, Cyprus; 9 Microbiology Department, Limassol General Hospital, Limassol 4131, Cyprus; 10 Medical School, University of Nicosia, Nicosia 2417, Cyprus; 11 Eurofins Genomics Sequencing Europe, 85560 Ebersberg, Germany; 12 S.C.I.N.A Bioanalysis Sciomedical Centre Ltd., Limassol 4040, Cyprus; 13 Cyprus Academy of Sciences, Letters, and Arts, 60-68 Phaneromenis Street, Nicosia 1011, Cyprus

**Keywords:** SARS-CoV-2, COVID-19, Cyprus, molecular epidemiology

## Abstract

The emergence of severe acute respiratory syndrome coronavirus 2 (SARS-CoV-2) in December 2019 resulted in the coronavirus disease 2019 (COVID-19) pandemic, which has had devastating repercussions for public health. Over the course of this pandemic, the virus has continuously been evolving, resulting in new, more infectious variants that have frequently led to surges of new SARS-CoV-2 infections. In the present study, we performed detailed genetic, phylogenetic, phylodynamic and phylogeographic analyses to examine the SARS-CoV-2 epidemic in Cyprus using 2352 SARS-CoV-2 sequences from infected individuals in Cyprus during November 2020 to October 2021. During this period, a total of 61 different lineages and sublineages were identified, with most falling into three groups: B.1.258 & sublineages, Alpha (B.1.1.7 & Q. sublineages), and Delta (B.1.617.2 & AY. sublineages), each encompassing a set of S gene mutations that primarily confer increased transmissibility as well as immune evasion. Specifically, these lineages were coupled with surges of new infections in Cyprus, resulting in the following: the second wave of SARS-CoV-2 infections in Cyprus, comprising B.1.258 & sublineages, during late autumn 2020/beginning of winter 2021; the third wave, comprising Alpha (B.1.1.7 & Q. sublineages), during spring 2021; and the fourth wave, comprising Delta (B.1.617.2 & AY. sublineages) during summer 2021. Additionally, it was identified that these lineages were primarily imported from and exported to the UK, Greece, and Sweden; many other migration links were also identified, including Switzerland, Denmark, Russia, and Germany. Taken together, the results of this study indicate that the SARS-CoV-2 epidemic in Cyprus was characterized by successive introduction of new lineages from a plethora of countries, resulting in the generation of waves of infection. Overall, this study highlights the importance of investigating the spatiotemporal evolution of the SARS-CoV-2 epidemic in the context of Cyprus, as well as the impact of protective measures placed to mitigate transmission of the virus, providing necessary information to safeguard public health.

## 1. Introduction

Severe acute respiratory syndrome coronavirus 2 (SARS-CoV-2), which emerged in December 2019 in Wuhan, China, is the causative agent of coronavirus disease 2019 (COVID-19) [1,2]. The virus rapidly spread globally, and by March 2020, the World Health Organization (WHO) declared COVID-19 a pandemic [3]. The number of SARS-CoV-2 infections has been increasing every year since, with 9927 cases by January 2020, 84.34 million by January 2021, and 378.90 million cases by January 2022 [4]. As of November 2022, more than 638,110,778 total cases and more than 6,620,904 deaths around the world due to SARS-CoV-2 have been documented; in Cyprus, which is the focus of this study, there have been more than 610,023 total cases and 1218 deaths [5,6,7]. These data emphasize the immense scale of SARS-CoV-2 transmission over time, around the world and in Cyprus.

As a result of high transmission and replication rates, along with other evolutionary pressures, such as the host’s immune system, SARS-CoV-2 has been accumulating mutations over the course of the pandemic [8,9,10,11]. Consequently, genetically similar yet distinct variants and lineages, each harboring characteristic mutations, have emerged [11,12]. Some of these mutations are reported to increase transmissibility, immune evasion and virulence [11,13,14,15]. Some variants with such high-risk characteristics have been termed variants of concern (VOC) by the World Health Organization (WHO) throughout the course of the pandemic [16,17].

The first four VOCs were Alpha (WHO Greek alphabet nomenclature) (B.1.1.7, Pango lineage classification system), Beta (B.1.351), Gamma (P.1) and Delta (B.1.617.2), which were identified and designated during late 2020 to early 2021 [16,17]. Specifically, Alpha and Beta were designated as VOCs by the WHO on 18 December 2020, Gamma on 11 January 2021, and Delta (B.1.617.2) on 11 May 2021 [16]. However, the earliest documented isolates of these VOCs were found months before their VOC designation, with the first detection of Beta as early as May 2020, Alpha in September 2020, Delta in October 2020, and Gamma in November 2020 [16]. Furthermore, the earliest documented records of these VOCs were reported in various geographic areas, with Alpha first being detected in the United Kingdom (UK), Beta in South Africa, Gamma in Brazil, and Delta in India [16,17,18,19].

Irrespective of origin, these VOCs quickly outcompeted other circulating lineages and began spreading around the world [17,20,21,22]. Although these four referenced VOCs exhibited increased transmissibility, the Beta and Gamma variants were initially associated with a higher degree of concern due to mutations in the spike gene, which were reported to confer reduced susceptibility to antibodies and to decrease vaccine efficacy [11,22]. However, these variants were not transmitted to the same degree as Alpha and Delta [22,23]. Moreover, Delta outcompeted Alpha, becoming the dominant variant worldwide, with reported transmissibility 40–60% higher than that of Alpha [24,25]. As of 7 June 2022, the WHO declared all of the aforementioned four VOCs to be out of circulation [16]. The only currently circulating VOC and the fifth variant to have been designated by the WHO on 26 November 2021 is Omicron, which includes B.1.1.529, BA.1, BA.2, BA.3, BA.4, BA.5 and descendent lineages [16]. The earliest documented isolates of Omicron were detected in November 2021 in multiple countries (South Africa and Botswana), and since its emergence, it has rapidly spread to become the dominant variant worldwide [16,23,26].

Numerous lineages have begun and ceased to circulate since the start of the pandemic. Cyprus was no exception with regard to this pattern of lineage replacement, with certain lineages causing surges of new infections, reaching a distinct peak and then a decline; this peaks-and-valleys pattern is referred to as ‘waves’ [27,28]. Specifically, in our previous study covering the period of April 2020 to January 2021, we determined that despite polyphyletic infection in Cyprus with 34 different lineages, there were three consecutive waves characterized by specific lineages [29]: B.1.1, specifically B.1.1.29, comprising the first wave with sequences from April 2020 to June 2020; B.1.258 & sublineages comprising the second wave, with sequences from September 2020 to January 2021; and Alpha (B.1.1.7 & Q. sublineages), comprising the beginning of the third wave, with sequences from December 2020 until the end of the sampling period in January 2021 [29].

In this study, we performed detailed genomic epidemiology analyses to study the SARS-CoV-2 epidemic in Cyprus for the period of November 2020 to October 2021 and to discern the most prevalent lineages circulating in the country, the mutations they harbor, and the spatiotemporal characteristics of those lineages. The results revealed 61 different lineages and sublineages, with the majority falling into three groups: B.1.258 & sublineages, Alpha (B.1.1.7 & Q. sublineages), and Delta (B.1.617.2 & AY. sublineages). B.1.258 & sublineages comprised the second wave, with sequences from November 2020 to March 2021; Alpha (B.1.1.7 & Q. sublineages) comprised the third wave from December 2020 to July 2021; and Delta (B.1.617.2 & AY. sublineages) comprised the fourth wave from April 2021 and continued until the end of the sampling period in October 2021. Additionally, it was identified that most SARS-CoV-2 imports and exports were from and to the UK, Greece, and Sweden, though many other migration links were found, such as Switzerland, Denmark, Russia, and Germany. This study demonstrates the strong value of elucidating the impact of SARS-CoV-2 infection in Cyprus while additionally showing that waves are predominantly influenced by the introduction of new, antigenically different, and typically more transmissible lineages.

## 2. Materials and Methods

### 2.1. Sequences Used in the Study

The sequences used in this study were derived from the molecular epidemiological surveillance study performed by the Laboratory of Biotechnology and Molecular Virology at the University of Cyprus (BMV UCY) in collaboration with the Cyprus Ministry of Health and members of the Cypriot Comprehensive Molecular Epidemiological Study on SARS-CoV-2 (COMESSAR) Network. As part of this collaboration, the BMV UCY retrospectively received 2365 whole-genome SARS-CoV-2 sequences from infected individuals in Cyprus from 04 November 2020 to 08 October 2021 from the Cyprus Ministry of Health.

From the initial 2365 SARS-CoV-2 sequences, 13 were excluded due to missing data (collection dates) and poor sequencing quality (too many missing/ambiguous bases) and were therefore not able to be classified into lineages using the Pangolin webtool (https://pangolin.cog-uk.io/, accessed on 7 May 2022) [30]. The remaining 2352 whole-genome SARS-CoV-2 sequences were included in the analysis. The samples of these 2352 sequences originated from 10 facilities in Cyprus where SARS-CoV-2 testing was performed: 1150 sequences from the Cyprus Institute of Neurology and Genetics (CING); 355 sequences from the Limassol General Hospital; 291 sequences from the Nicosia General Hospital; 188 sequences from the Larnaca General Hospital; 157 sequences from the NIPD Genetics; 120 sequences from the S.C.I.N.A. Bioanalysis Sciomedical Centre Ltd.; 39 sequences from Mygene Molecular Diagnostics Ltd.; 31 sequences from Synlab Cyprus; 14 sequences from Archbishop Makarios III Hospital; and 7 sequences from Tymvios Medical Labs. Sequencing of these samples was performed primarily by the laboratory of Eurofins Genomics Sequencing Europe, which sequenced 1989 of the abovementioned samples; the S.C.I.N.A. Bioanalysis Sciomedical Centre Ltd. sequenced 272 samples, and NIPD Genetics sequenced 91. Bioethical approval (EEBK 21.1.04.43.01) was provided by the Cyprus National Bioethics Committee for analyses of these sequences, and to ensure anonymity, all sequences were coded with a new laboratory code (double coded) upon being received from the BMV UCY lab. The use of the sequences was in accordance with the relevant guidelines and regulations of the Cyprus National Bioethics Committee.

### 2.2. Sample Collection, RNA Extraction and SARS-CoV-2 Real-Time RT–PCR

Nasopharyngeal and/or oropharyngeal swab samples in transport medium were collected or received by each facility for diagnostic purposes. Specifically, Limassol General Hospital and Archbishop Makarios III Hospital used swabs and transport medium from Jiangsu Yuli Medical Instrument, Shengao, China. Larnaca General Hospital, S.C.I.N.A. Bioanalysis Sciomedical Centre Ltd., and Tymvios Medical Labs used swabs and transport medium from Biocomma Limited, Guangdong, China. NIPD Genetics used swabs from Puritan, Guilford, ME, USA and transport medium from Thermo Scientific, Waltham, MA, USA. Mygene Molecular Diagnostics Ltd. used swabs and transport medium from BIOBASE Shandong, China. Synlab Cyprus used swabs and transport medium from Dakewe BioSci, Sacramento, CA, USA. Nicosia General Hospital, which performed both conventional and fully automated protocols, used swabs and transport medium from the following: 1. Bioprepare, Athens, Greece; 2. KANG JIAN Medical Instrument, Taizhou, China; 3. AMV Healthcare Ltd., Nicosia, Cyprus; 4. Copan, Brescia, Italy (only used for the automated protocol). CING received nasopharyngeal swab samples in transport medium from COVID-19 Public Health Clinics (Nicosia, Limassol, Larnaca, Paphos, Famagusta).

Following sample collection, RNA extraction was performed. Regarding extraction kits, CING used MagMAX™ Viral/Pathogen Nucleic Acid Isolation Kit (Applied Biosystems, Waltham, MA, USA). S.C.I.N.A. Bioanalysis Sciomedical Centre Ltd. and Tymvios Medical Labs used Mag-Bind^®^ Viral RNA Xpress Kit (Omega Biotek, Norcross, GA, USA) or MagMAX™ Viral/Pathogen II (MVP II) Nucleic Acid Isolation Kit (Applied Biosystems, Waltham, MA, USA). NIPD Genetics, Larnaca, Nicosia (conventional protocol) and Limassol General Hospitals used TANBead^®^ OptiPure Viral Auto Plate Kit (TANBead, Taoyuan, Taiwan). Mygene Molecular Diagnostics Ltd. employed Mag-Bind^®^ Viral DNA/RNA 96 Kit (Omega Biotek, Norcross, GA, USA) and Synlab Cyprus used Mag-Bind^®^ Viral RNA Xpress Kit (Omega Biotek, Norcross, GA, USA). Regarding the equipment used for RNA extraction, CING, S.C.I.N.A. Bioanalysis Sciomedical Centre Ltd., Mygene Molecular Diagnostics Ltd., Tymvios Medical Labs and Synlab Cyprus utilized KingFisher™ Flex Purification System (Thermo Fisher Scientific, Waltham, MA, USA). NIPD Genetics, Larnaca and Limassol General Hospitals used the SLA-32 instrument (TANBead, Taoyuan, Taiwan) and the Nicosia General Hospital (conventional protocol) TANBead Nucleic Acid Extractor (TANBead, Taoyuan, Taiwan).

Identification of SARS-CoV-2-positive samples was performed using real-time RT–PCR. Specifically, regarding the kits used for real-time RT–PCR, CING employed the TaqPath COVID-19 CE-IVD RT–PCR kit (Thermo Fisher Scientific, Waltham, MA, USA) (ORF1ab, S and N genes). SARS-CoV-2 Real-TM Kit (Sacace Biotechnologies, Como, Italy) (E and N genes) or VIASURE SARS-CoV-2 Real Time Detection Kit (CerTest Biotec, Zaragosa, Spain) (ORF1ab and N genes) was used by Limassol and Larnaca General Hospitals. Nicosia General Hospital also used VIASURE SARS-CoV-2 Real Time PCR Detection Kit (CerTest Biotec, Zaragosa, Spain) (ORF1ab and N genes) for their conventional protocol. For the automated protocol of the Nicosia General Hospital, which includes both RNA extraction and real-time RT–PCR, the Xpert^®^ Xpress SARS-CoV-2 cartridge (Cepheid, Solna, Sweden) (N (N2), and E gene) with GeneXpert^®^ Instrument Systems (Cepheid, Sunnyvale, CA, USA) was utilized. Archbishop Makarios III Hospital also employed the above automated protocol but used the Xpert^®^ Xpress SARS-CoV-2 cartridge (Cepheid, Solna, Sweden) or Xpert^®^ Xpress SARS-CoV-2/Flu/RSV cartridge (Cepheid, Solna, Sweden) (N (N2) and E gene). NIPD Genetics used SensiFAST Probe No-ROX One-Step Kit (Meridian Bioscience, Cincinnati, OH, USA) and two primer/probe sets targeting the SARS-CoV-2 N gene (N1 and N2) (Integrated DNA Technologies, Coralville, IA, USA). S.C.I.N.A. Bioanalysis Sciomedical Centre Ltd. and Tymvios Medical Labs implemented Fosun COVID-19 RT–PCR Detection Kit (Shanghai Fosun Long March Medical Science Co. LTD, Shanghai, China) (ORF1ab, E and N gene); Mygene Molecular Diagnostics Ltd. used AgPath-ID™ One-Step RT–PCR Reagents (Thermo Scientific, Waltham, MA, USA) and two primer/probe sets targeting a conserved region of the SARS-CoV-2 E and RdRP genes (Charité/Berlin protocol) (Integrated DNA Technologies, Coralville, IA, USA). SARS-CoV-2 ELITe MGB^®^ Kit (ElitechGroup, Puteaux, France) (Orf-8 and RdRp genes) was used by Synlab Cyprus. Regarding the equipment utilized for real-time RT–PCR, CING, S.C.I.N.A. Bioanalysis Sciomedical Centre Ltd. and Synlab Cyprus used the QuantStudio 5 Real-Time PCR system (Applied Biosystems, Waltham, MA, USA). Mygene Molecular Diagnostics Ltd., and Limassol and Larnaca General Hospitals used the Qiagen Rotor-Gene Q MDx 5plex platform (Qiagen, Hilden, Germany). Nicosia General Hospital used the Qiagen Rotor Gene Q instrument (Qiagen, Hilden, Germany). NIPD Genetics used the Bio-Rad CFX 384 system (Bio-Rad, Hercules, CA, USA) and Tymvios Medical Labs used Heal Force 960B Real-Time PCR System (Heal Force Bio-Medich Tech Holdings LTD, Hong Kong, China).

### 2.3. Next-Generation Sequencing (NGS)

#### 2.3.1. NGS by Eurofins Genomics Sequencing Europe

RNA samples from SARS-CoV-2-infected individuals were transcribed into complementary DNA (cDNA) using LunaScript RT SuperMix (NEB) (New England Biolabs, Ipswich, MA, USA). Positive and negative controls were included in each run. The cDNA was then used for enrichment of the viral genome with ARTIC V3 [31] primers (or updated versions thereof) covering the full 29.9-kb viral genome. Amplification was carried out using NEBNext Ultra II Q5 Master Mix (New England Biolabs, Ipswich, MA, USA). The ARTIC amplicons of SARS-CoV-2-infected samples and corresponding positive and negative controls were converted into sequencing libraries, and sample-specific index codes were attached by PCR prior to pooling. The pool was then quantified and analyzed regarding the size of the pool prior to sequencing. Sequencing was performed using an Illumina NovaSeq6000 (Illumina Inc., San Diego, CA, USA) in 150-bp paired-end read mode. All sequencing was performed using original chemistry provided by Illumina (Illumina Inc., San Diego, CA, USA).

Demultiplexed sequencing data were analyzed using an in-house developed pipeline (Eurofins Genomics Covid Pipeline v.1.0 or updated versions thereof). In brief, the primers used for amplicon generation were scanned and removed from the sequencing reads, followed by removal of low-quality bases (<Q30) and reads shorter than 50 nt in length. The remaining high-quality reads were aligned to the SARS-CoV-2 reference genome (isolated Wuhan-Hu-1, NCBI Reference Sequence: NC_045512.2, GenBank: MN908947.3) using the BWA-MEM algorithm [32]. The read alignments were further refined by retaining only full-length primary alignments, followed by removal of redundant alignments using the Gencore algorithm [33]. High-quality cleaned read alignments were then used to generate a final consensus sequence using the Consensusfixer algorithm [34] with at least 15 supporting reads at each position. Base positions that had <15× or zero coverage were filled with Ns.

#### 2.3.2. NGS by NIPD Genetics and S.C.I.N.A. Bioanalysis Sciomedical Centre Ltd.

The NGS methodology used by NIPD Genetics and S.C.I.N.A. Bioanalysis Sciomedical Centre Ltd., employed COVIDSeq Assay (Illumina Inc., San Diego, CA USA) with ARTIC V.3 PCR primers, which were described in detail in our previous publication (Section 2.3. Next Generation Sequencing (NGS)) [29]. However, for S.C.I.N.A. Bioanalysis Sciomedical Centre Ltd., the indexing used was IDT for Illumina-PCR Indexes Sets 1–4 (384 Indexes) (part of the Illumina COVIDSeq RUO); the NextSeqTM 550Dx instrument (Illumina Inc., San Diego, USA) was employed, with samples sequenced at a read length of 2 × 151 bp. Additionally, the methodology used by S.C.I.N.A. Bioanalysis Sciomedical Centre Ltd. for demultiplexing, processing and assembling of the raw sequencing reads to make the final consensus involved DRAGEN COVIDSeq Test (RUO) app version 1.3.0 (Illumina Inc., San Diego, CA, USA).

### 2.4. Bioinformatic Analysis

#### 2.4.1. Lineage Classification

Lineage classification was performed using Pangolin Webtool (Pangolin-data version v1.8, Pangolin version 4.0.6) (https://pangolin.cog-uk.io/, accessed on 7 May 2022) [30].

#### 2.4.2. Mutation Calling

Mutations in the sequences in this study were identified using Nextclade Webtool Web version 1.14.1 (https://clades.nextstrain.org/Nextclade accessed on 7 May 2022) [35]. To avoid misinterpretation of mutations from data that may result from sequencing and assembly artifacts, such as an excessive amount of ambiguous nucleotides, missing data, frameshifts and stop codons, the mutation analysis was based on the 1759 sequences that passed a stricter quality control threshold [35].

#### 2.4.3. Dataset Compilation

Phylogenetic analyses involved the datasets for B.1.258 & sublineages, Alpha (B.1.1.7 & Q. sublineages) and Delta (B.1.617.2 & AY sublineages). To alleviate computational burden, these lineages were analyzed separately, and their delineation was performed according to the same strategy as in our previous study [29]. Specifically, all newly generated near whole genomes were multiple-aligned using the MAFFT v.7.475 multiple sequence alignment program [36]. The alignments were visually inspected and manually edited with the AliView v.1.26 algorithm [37]. A maximum likelihood (ML) tree was then constructed from the edited alignment using IQtree v.2.1.2 software [38], with branch support estimated through the SH-like approximate likelihood ratio test (SH-aLRT) [39] and ultrafast bootstrap (UFB) procedure [40]. An SH-aLRT and UFB threshold of 90 and 100, respectively, was used to identify the lineages of interest.

To infer time-scaled phylogeographic histories, the genomes from the lineages were complemented with publicly available SARS-CoV-2 genomes. To this end, available complete genomes were downloaded from GISAID on 30 March 2022 (9,792,312 sequences) [41,42]. For each lineage, the 25 genomic sequences most similar to each of the newly generated near-complete genomes were selected using National Center for Biotechnology Information Basic Local Alignment Search Tool (NCBI BLASTn v.2.11.0+) [43], and all duplicate hits were removed. After alignment of these sequences with MAFFT v.7.475 [36] and editing with the AliView v.1.26 algorithm [37], a lineage-specific ML tree was produced using IQtree [38]. This served to further reduce the dataset size by replacing sequences sampled in the same country that cluster together with perfect UFB support by a randomly selected sequence thereof. This procedure yielded datasets with 887 (B.1.258 & sublineages), 3748 (B.1.1.7 & Q. sublineages) and 6798 (B.1.617.2 & AY. sublineages) genomes.

#### 2.4.4. Time-Scaled Phylogeographic Inference

The size of the Alpha (B.1.1.7 & Q. sublineages) and Delta (B.1.617.2 & AY sublineages) datasets precluded integrated analysis to jointly infer epidemic relationships and migration history. We first generated a time-scaled ML tree using IQtree [38] and LSD2 in R (Rlsd2: R-wrapper for LSD2. R package version 1.10) [44]. For the latter, the rate of evolution was set to 0.0008 s/s/y [45], and taxa with z scores > 3 were considered outliers and removed. To account for the branching uncertainty in phylogeographic reconstructions, polytomies were randomly resolved 1000 times, imposing a small nonzero minimum branch length. The resulting collection of bifurcating trees served as an empirical tree distribution [46] to estimate migration history using an asymmetric discrete phylogeographic model that incorporated a model-averaging procedure (the Bayesian stochastic search variable selection procedure [47]) to identify subsets of migration flows that adequately explain the diffusion process [48], as implemented in BEAST v. 1.10 [49]. Only migration links with Bayes factor support ≥ 5 were reported. The expected number and timing of transitions between locations was estimated using stochastic mapping techniques [50]. For B.1.258 & sublineages, the reported country of sampling was used as the location state. The size and complexity of the Alpha (B.1.1.7 & Q. sublineages) and Delta (B.1.617.2 & AY sublineages) datasets, however, led us to group genomes from countries with fewer than 20 genomes in accordance with the United Nations (UN) geographical subregion. This reduced the number of location states from 66 to 35 for Alpha (B.1.1.7 & Q. sublineages) and from 81 to 40 for Delta (B.1.617.2 & AY sublineages).

#### 2.4.5. Time-Scaled Phylogenetic Inference

Time-scaled phylogenies from large clades that represent within-Cyprus circulation were estimated through Markov chain Monte Carlo (MCMC) simulation, as implemented in BEAST 1.10 software [49]. To this end, the substitution process was modeled using an HKY85 model with gamma-distributed among-site rate variation [51,52]. A strict clock was applied with a normal distribution with a mean of 0.0008 s/s/y and a standard deviation of 0.001 as the evolutionary rate parameter [45]. The skygrid model [53] was specified as a flexible tree prior, with change points set at 2-week intervals.

### 2.5. Calculations and Figure Information

**Figure 1:**
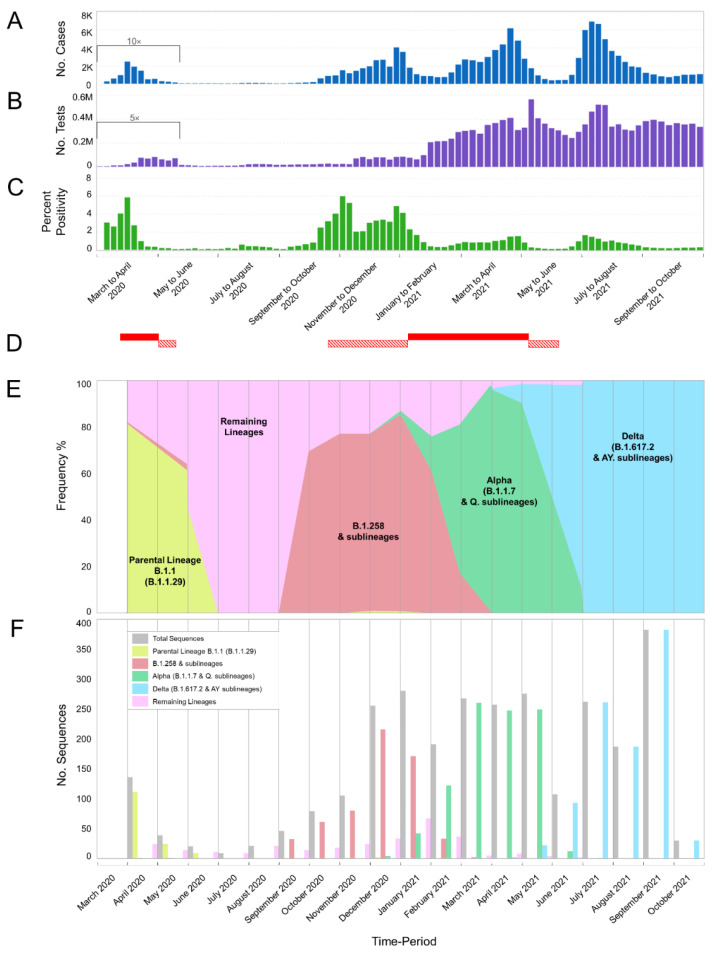
Cases, tests, percent positivity, and most prevalent lineages of SARS-CoV-2 infection in Cyprus from March 2020 until October 2021. The present study focuses on the period of November 2020 to October 2021; our previous study covered the period of April 2020 to January 2021 [29] and overlaps from November 2020 to January 2021. The previous period of April 2020 to January 2021 was included for continuity purposes as well as to show the progression of SARS-CoV-2 infection in Cyprus. (**A**) The number of positive SARS-CoV-2 cases per week. Values of positive cases are depicted with dark blue columns; values underneath the black bracket, covering the period from 01 March 2020 to 17 May 2020, were multiplied by 10 to enhance their visibility. (**B**) The total number of SARS-CoV-2 tests (PCR and rapid) performed per week in Cyprus. PCR and rapid tests were combined for the purposes of this figure. The values of the tests are depicted with purple columns, and the values underneath the black bracket, covering the period from 01 March 2020 to 17 May 2020 were multiplied by 5 to enhance their visibility. (**C**) The calculated percent positivity of SARS-CoV-2 testing per week. Values of percent positivity are depicted with green columns. (**D**) The red-filled horizontal rectangles indicate periods in which Cyprus was under lockdown; horizontal rectangles with diagonal red lines indicate periods in which Cyprus was under partial lockdown. The first lockdown was from 24 March 2020 to 03 May 2020, and the second was from 10 January 2021 to 09 May 2021. The first partial lockdown was from 04 May 2020 to 20 May 2020, the second was from 23 October 2020 to 09 January 2021, and the third was from 10 May 2021 to 10 June 2021 (information provided by the Ministry of Health). Brackets underneath (**A**–**C**) group the weeks into approximately 2-month periods. (**E**,**F**) show the frequency (proportion) and number of sequences of the most prevalent lineages in Cyprus per month, respectively. Specifically, sequences for lineages B.1.1.29 (Parental Lineage B.1.1), B.1.258 & sublineages, Alpha (B.1.1.7 & Q. sublineages) and Delta (B.1.617.2 & AY. sublineages) are shown in bright-green, red, green, and light blue, respectively. Numbers of sequences in the “Remaining Lineages” are shown in pink, as calculated by excluding the monthly sequences of the lineages B.1.1.29 (Parental lineage B.1.1), B.1.258 & sublineages (B.1.258.17 and B.1.258.22), Alpha (B.1.1.7 & Q. sublineages) and Delta (B.1.617.2 & AY. sublineages) lineages from the total number of sequences shown in gray. There were no available sequencing data for the month of March 2020.

**Figure 2:**
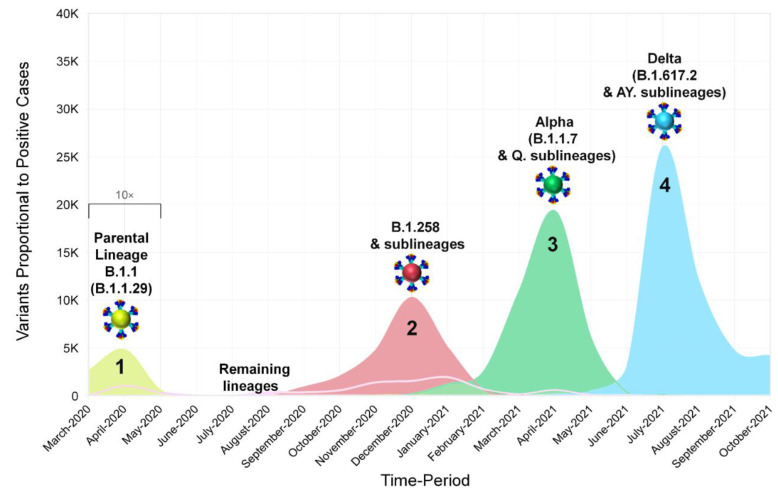
The progression and waves of SARS-CoV-2 infection in Cyprus from March 2020 until October 2021. Data from our previous work covering the period of April 2020 to January 2021 [29] were included for continuity purposes as well as to show the development of SARS-CoV-2 infection in Cyprus. In this figure, the number of positive SARS-CoV-2 cases per month reported in Cyprus from March 2020 until October 2021 is shown proportionally to the most prevalent SARS-CoV-2 variants (see Figure 1A,E,F). Sequences of B.1.1.29 (Parental Lineage B.1.1), B.1.258 & sublineages, Alpha (B.1.1.7 & Q. sublineages) and Delta (B.1.617.2 & AY. sublineages), and the remaining lineages, as shown in the smoothed line chart, are depicted in bright-green, red, green, light-blue, and pink, respectively. Values underneath the black bracket (March 2020–May 2020) were multiplied by 10 to enhance their visibility.

The data for Figure 1A,B and Figure 2 were obtained from the Cyprus Ministry of Health, the Press and Information Office and the KIOS Research and Innovation Center of Excellence (KIOS CoE) that operates within the University of Cyprus, and were processed to calculate the percent positivity for Figure 1C [7,54,55]. Specifically, the calculations performed for the percent positivity of Figure 1C were performed by dividing the number of positive SARS-CoV-2 cases per week by the total number of SARS-CoV-2 tests per week and then obtaining the percentage [54]. Regarding the calculations for Figure 2, the number of positive SARS-CoV-2 cases per month reported in Cyprus from March 2020 until October 2021 was indicated proportionally to the most prevalent SARS-CoV-2 variants. Additionally, for Figure 2, the illustration of the spike proteins on the colored virions underneath the names of each lineage, as also seen in figures below, was produced by PyMol (Version 2.4.1, Schrödinger, LLC, https://www.pymol.org, accessed on 18 February 2021) and is based on data derived and adapted from Protein Data Bank entry 6XEY [56,57], as well as other sources used to outline spike protein domains [58,59,60,61,62,63,64,65,66].

## 3. Results

### 3.1. The Appearance of Lineages and the Waves of SARS-CoV-2 Infection in Cyprus

For this study, 2352 SARS-CoV-2 whole-genome sequences obtained in Cyprus were analyzed for the period from November 2020 to October 2021. The lineage classification analysis revealed a plethora of lineages, with 61 different lineages being identified over the course of this study period (Table 1). For the first three-month period (November 2020–January 2021) (Table 1), the most prevalent lineages were B.1.258 (66.75%, 263/394 sequences) and its sublineages (1.27%, 5/394), accounting for a total of 268/394 (68.02%) sequences from this period. The Alpha variant (B.1.1.7) started to rise in prevalence during this period, accounting for 9.14% (36/394) sequences. This 3-month period (November 2020–January 2021) overlapped with our previous molecular epidemiology study in Cyprus from April 2020 to January 2021 (lineages classified using Pangolin v.2.3.3) [29], with the most prevalent lineage being B.1.258 for both studies (Figure 1E,F and Figure 2).

The first three-month period (November 2020–January 2021) coincides with the second wave of SARS-CoV-2 in Cyprus, with the majority of sequences being B.1.258 & sublineages (Figure 1A,E,F and Figure 2). The prevalence of the B.1.258 and sublineage sequences peaked in December 2020 and declined by February 2021, with the last identification in March 2021 (Figure 1E,F and Figure 2). Of note, similar to the first wave, the initial period of the second wave was characterized by a substantially increasing rate of positivity (Figure 1A–C).

The next three-month period (February 2021–April 2021), encompassing the initial period of the third wave, coincides with a marked shift in prevalence between B.1.258 & sublineages and the Alpha variant (B.1.1.7 & Q. sublineages). During this period, B.1.258 and its sublineages declined in prevalence by 4.9% (35/715), with no sequences detected by April 2021 (Table 1, Figure 1E,F and Figure 2). The Alpha variant (B.1.1.7) (87.69%, 627/715) and Q. sublineages (0.28%, 2/715) became dominant, reaching a prevalence of 87.97% (629/715) (Table 1). During this period, there was also an increase in percent positivity (Figure 1B,C), though it was less pronounced with respect to the early phases of the first and second waves of infections. The demise of the third wave coincides with the declining prevalence of the Alpha variant during June 2021, and it was last identified in July 2021 (Figure 1A and Figure 2). Around this period, the first Delta (B.1.617.2) sequences were detected at low prevalence during the February 2021 to April 2021 period, with 2/715 (0.28%) sequences in April 2021 (Table 1 and Figure 1E,F). Additionally, 7/715 (0.98%) sequences of the Eta (B.1.525) variant (previously circulating variant of interest [16]) and 1/715 (0.14%) sequence of the B.1.1.523 variant (previously circulating variant under monitoring [16]) were identified during this three-month period (Table 1).

By the next three-month period (May 2021–July 2021), the Delta variant (B.1.617.2) at 1.55% (10/644) and AY. sublineages at 56.86% (366/644) became dominant at 58.41% (376/644), whereas the Alpha variant prevalence declined to 40.69% (262/644) (Table 1). This period marked the appearance of the fourth wave in Cyprus, which was driven almost uniquely by the Delta variant (Figure 2). Once more, the upsurge of SARS-CoV-2 infections was accompanied by an increase in percent positivity (Figure 1B,C). At the start of the fourth wave, some non-Delta variants were detected, including one sequence (0.16%; 1/644) of the Beta variant (B.1.351). Delta and its AY. sublineages gained total dominance in the following months (Table 1 and Figure 1).

In summary, by the end of the sampling period, there were three waves of infections, each characterized by particular dominant groups of lineages. These lineages were (in order of chronological appearance): B.1.258 & sublineages (12.88%, 303/2352); Alpha (B.1.1.7 & Q. sublineages) (39.14%, 927/2352); and Delta (B.1.617.2 & AY. sublineages) (41.54%, 977/2352) (Table 1).

**Table 1:** SARS-CoV-2 lineages identified from 2352 sequences in Cyprus from November 2020 to October 2021.

Time Period	Nov 2020–Jan 2021	Feb–Apr 2021	May–July 2021	Aug–Oct 2021	Total
Lineage	Νumber of Sequences per Lineage (%)	Νumber of Sequences per Lineage (%)	Νumber of Sequences per Lineage (%)	Νumber of Sequences per Lineage (%)	Νumber of Sequences per Lineage (%)
AD.2	1 (0.25)	-	-	-	1 (0.04)
AY.1	-	-	-	1 (0.17)	1 (0.04)
AY.4	-	-	5 (0.78)	71 (11.85)	76 (3.23)
AY.4.2	-	-	-	2 (0.33)	2 (0.09)
AY.4.3	-	-	-	2 (0.33)	2 (0.09)
AY.4.4	-	-	-	5 (0.83)	5 (0.21)
AY.4.5	-	-	-	1 (0.17)	1 (0.04)
AY.5	-	-	-	1 (0.17)	1 (0.04)
AY.6	-	-	-	2 (0.33)	2 (0.09)
AY.7	-	-	2 (0.31)	-	2 (0.09)
AY.7.2	-	-	-	2 (0.33)	2 (0.09)
AY.9	-	-	1 (0.16)	8 (1.34)	9 (0.38)
AY.9.2	-	-	1 (0.16)	5 (0.83)	6 (0.26)
AY.13	-	-	-	1 (0.17)	1 (0.04)
AY.23	-	-	1 (0.16)	-	1 (0.04)
AY.25.1	-	-	-	2 (0.33)	2 (0.09)
AY.34.1	-	-	-	2 (0.33)	2 (0.09)
AY.36	-	-	-	1 (0.17)	1 (0.04)
AY.42	-	-	-	1 (0.17)	1 (0.04)
AY.43	-	-	1 (0.16)	46 (7.68)	47 (2.00)
AY.44	-	-	-	2 (0.33)	2 (0.09)
AY.46	-	-	1 (0.16)	-	1 (0.04)
AY.46.6	-	-	-	3 (0.50)	3 (0.13)
AY.60	-	-	106 (16.46)	62 (10.35)	168 (7.14)
AY.92	-	-	-	1 (0.17)	1 (0.04)
AY.98	-	-	2 (0.31)	1 (0.17)	3 (0.13)
AY.98.1	-	-	-	9 (1.50)	9 (0.38)
AY.103	-	-	-	1 (0.17)	1 (0.04)
AY.116	-	-	-	1 (0.17)	1 (0.04)
AY.120	-	-	-	3 (0.50)	3 (0.13)
AY.122	-	-	246 (38.20)	305 (50.92)	551 (23.43)
AY.125	-	-	-	3 (0.50)	3 (0.13)
AY.126	-	-	-	5 (0.83)	5 (0.21)
AY.128	-	-	-	5 (0.83)	5 (0.21)
B.1	5 (1.27)	-	-	-	5 (0.21)
B.1.1.7	36 (9.14)	627 (87.69)	261 (40.53)	-	924 (39.29)
B.1.1.25	8 (2.03)	2 (0.28)	-	-	10 (0.43)
B.1.1.219	1 (0.25)	-	-	-	1 (0.04)
B.1.1.312	3 (0.76)	-	-	-	3 (0.13)
B.1.1.317	1 (0.25)	1 (0.14)	-	-	2 (0.09)
B.1.1.487	2 (0.51)	-	-	-	2 (0.09)
B.1.1.523	-	1 (0.14)	2 (0.31)	-	3 (0.13)
B.1.36.31	1 (0.25)	-	-	-	1 (0.04)
B.1.160	14 (3.55)	1 (0.14)	-	-	15 (0.64)
B.1.177	41 (10.41)	32 (4.48)	-	-	73 (3.10)
B.1.177.15	1 (0.25)	-	-	-	1 (0.04)
B.1.177.21	2 (0.51)	4 (0.56)	-	-	6 (0.26)
B.1.177.41	1 (0.25)	-	-	-	1 (0.04)
B.1.177.82	7 (1.78)	-	-	-	7 (0.30)
B.1.218	1 (0.25)	-	-	-	1 (0.04)
B.1.258	263 (66.75)	34 (4.76)	-	-	297 (12.63)
B.1.258.17	3 (0.76)	-	-	-	3 (0.13)
B.1.258.22	2 (0.51)	1 (0.14)	-	-	3 (0.13)
B.1.351	-	-	1 (0.16)	-	1 (0.04)
B.1.398	-	1 (0.14)	-	-	1 (0.04)
B.1.525	-	7 (0.98)	-	-	7 (0.30)
B.1.617.2	-	2 (0.28)	10 (1.55)	45 (7.51)	57 (2.42)
C.36.3	-	-	3 (0.47)	-	3 (0.13)
D.5	1 (0.25)	-	-	-	1 (0.04)
Q.6	-	1 (0.14)	-	-	1 (0.04)
Q.8	-	1 (0.14)	1 (0.16)	-	2 (0.09)
Total	394	715	644	599	2352

### 3.2. Spike Protein Mutations of the Most Prevalent Lineages/Variants in Cyprus

The most prevalent lineages that were identified in Cyprus during the period of November 2020 to October 2021, as mentioned above (Section 3.1), were B.1.258 and its sublineages (14.95%, 263/1759); Alpha (B.1.1.7) and Q. sublineages (39.28%, 691/1759); and Delta (B.1.617.2) and AY. sublineages (39.11%, 688/1759) (Tables S1 and S2). The analyses in this section focused on the S protein due to its important role in the transmissibility of the virus, as well as the fact that it is a target for vaccine design and diagnostics [67]. To identify the characteristic and most common mutations of these lineages, sequences were input into Nextclade Webtool [35], and the output was sorted to isolate S protein mutations (deletions and substitutions).

**Figure 3:**
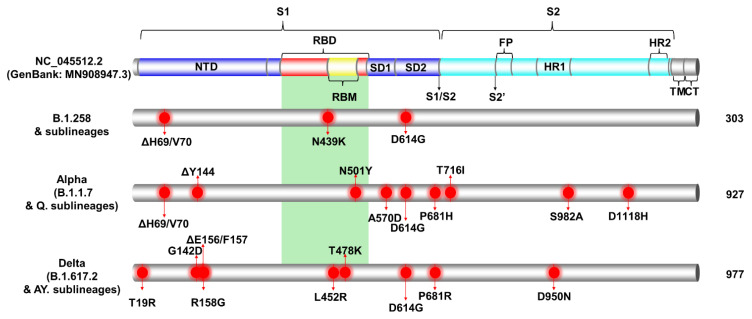
The most common S protein mutations of the most prevalent lineages identified from Cypriot SAR-CoV-2 sequences, samples of which were collected from November 2020 to October 2021. Specifically, this figure presents the most common S protein mutations of the most prevalent lineages of the second wave (B.1.258 & sublineages), the third wave (Alpha, B.1.1.7 & Q. sublineages), and the fourth wave (Delta, B.1.617.2 & AY. sublineages). The colored cylinder depicts key domains of the SARS-CoV-2 S protein (NC_045512.2) (GenBank: MN908947.3). NTD, N-terminal domain; RBD, receptor-binding domain (red); RBM, receptor-binding motif (yellow); SD1, subdomain 1; SD2, subdomain 2; FP, fusion peptide; S1, subunit 1 (blue); S2, subunit 2 (cyan); HR, heptad repeats; TM, transmembrane domain (gray); CT, cytoplasmic tail (gray). Cleavage sites are depicted with black arrows, S1/S2 and S2′, respectively. The green-highlighted region corresponds to the receptor-binding domain (RBD) [56,58,59,60,61,62,63,64,65,66]. Mutations were identified using the Nextclade webtool (https://clades.nextstrain.org, date accessed 9 May 2022) [35]. Red circles indicate locations of the most common mutations identified in all or nearly all of the sequences in a lineage. The total number of sequences per lineage in this study is indicated on the right-hand side of the figure.

The most common mutations of each lineage are depicted in Figure 3 and tabulated in Table S1. First, during the first wave in Cyprus (Figure 1 and Figure 2), which was described in our previous study [29], B.1.1 (specifically the B.1.1.29 lineage) was the most prevalent, and the sole most common mutation found in the S protein was D614G; thus, it is not included in Figure 3. The most common mutations for the most prevalent lineage in the second wave in Cyprus, B.1.258 and its sublineages, were ΔH69/V70, N439K and D614G. However, it is important to note that the two sublineages of B.1.258 found in this dataset, which are B.1.258.17 and B.1.258.22, harbor additional mutations in the S protein not found in parental B.1.258. Those mutations are L189F and V772I for B.1.258.17 and A67V for B.1.258.22 (Table S1).

Next, for the Alpha (B.1.1.7) variant and its Q. sublineages, which were predominant during the third wave, the most common mutations were ΔH69/V70, ΔY144, N501Y, A570D, D614G, P681H, T716I, S982A, and D1118H (Figure 3). The Q. sublineage of Alpha B.1.1.7, Q.8, was detected only once (1/1759, 0.06%), with the same number of S protein mutations as the parent lineage, B.1.1.7, in this dataset.

Last, for the Delta (B.1.617.2) variant and its AY. sublineages, which dominated the fourth wave, the most common mutations were T19R, G142D, ΔE156/F157, R158G, L452R, T478K, D614G, P681R, and D950N (Figure 3). Note that the mutations ΔE156/F157 and R158G can also be reported as E156G and ΔF157/R158 [68]. Unlike B.1.258 and Alpha (B.1.1.7), for which only a few sublineages were detected, 30 different sublineages of the Delta variant were identified in this dataset (Table S1). Contrary to B.1.258 and Alpha (B.1.1.7) that substantially outnumbered their sublineages, the parental lineage for Delta, B.1.617.2, was not the predominant lineage of this family (Table S1). In fact, the predominant sublineage of Delta was AY.122, at 52.91% (364/688), and that of parental B.1.617.2 was only 5.96% (41/688) of the Delta lineages in this dataset. As a result, analysis of the most common mutations is biased toward better represented sublineages. For example, the T95I S protein mutation is reported to be common in 13 of the 30 Delta AY. sublineages identified in this dataset according to the CoV-Spectrum website (https://cov-spectrum.org/, date accessed 30 May 2022) [23]; in other Delta AY. sublineages, it may be found in lower prevalence (Tables S1 and S2). Similarly, the A222V mutation is commonly found in AY. lineages such as AY.4.2 (Delta Plus) and AY.60; however, as they are represented in lower numbers or not the predominant Delta lineages (Table 1 and Table S1), their mutations were not as commonly represented in the whole Delta family in this dataset (Table S1) [69].

**Figure 4:**
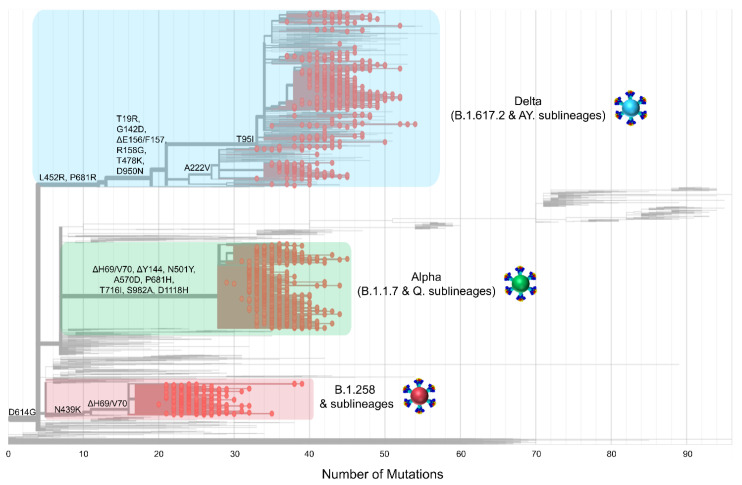
The phylogeny and S protein mutations of Cypriot SARS-CoV-2 sequences classified as B.1.258 & sublineages, Alpha (B.1.1.7 & Q. sublineages), and Delta (B.1.617.2 & AY. sublineages) from November 2020 to October 2021. The maximum likelihood tree was generated in Nextclade (https://clades.nextstrain.org, date accessed 05 August 2022 [35]) and highlights the emergence of mutations. Mutations that appear on the left-hand side of the figure emerged earlier during the pandemic; moving toward the right-hand side are mutations that appeared as SARS-CoV-2 evolved and diverged into new lineages. The sequences of the most prevalent lineages of the second wave (B.1.258 & sublineages), third wave (Alpha, B.1.1.7 & Q. sublineages), and fourth wave (Delta, B.1.617.2 & AY. sublineages) are highlighted in red, green and blue rectangles, respectively. Red dots represent the sequences of B.1.258 & sublineages, Alpha (B.1.1.7 & Q. sublineages), and Delta (B.1.617.2 & AY. sublineages) used in this study.

The above shows that as the pandemic progressed, SARS-CoV-2 variants incorporated an increasingly diverse set of mutations (Figure 4). Lineages that evolved during the early stages of the pandemic (B.1 and its direct descendants) mostly carried the D614G mutation of the S protein [70]. As B.1 became the dominant lineage around the world, essentially all its descendant lineages, including those described in this section, retained the D614G mutation [70]. Other recurrent mutations that appeared later, such as the ΔH69/V70 deletions [71], were not found in all lineages; in this dataset, they are common for B.1.258 and the Alpha variant. However, the ΔH69/V70 deletions do not appear to have evolved from the same ancestor for B.1.258 and the Alpha variant (Figure 4 and [72]). Indeed, most mutations occurred in the S protein for the Alpha variant, and the entirety of mutations for B.1.258 are concentrated in the S1 subunit, specifically at the start of the N-terminal domain (NTD) and in the receptor-binding domain (RBD) (Figure 3). Similar to the Alpha variant and B.1.258, the Delta variant, which carries approximately the same number of mutations within the S protein as the Alpha variant in this dataset, shows the majority of those mutations concentrated in the S1 subunit (NTD and RBD) (Figure 3 and Figure 4). Furthermore, there are more mutations in the RBD, specifically in the receptor-binding motif (RBM), for Delta (L452R and T478K) than for Alpha (N501Y) or B.1.258 (N439K) (Figure 3). Interestingly, both the Alpha and Delta variant contain a mutation at amino acid P681 in the furin cleavage site that separates the S1 and S2 subunits (Figure 3). Finally, between B.1.258, the Alpha variant and the Delta variant, only the latter two harbor mutations found in the S2 subunit, namely T716I, S982A and D1118H for Alpha and D950N for Delta (Figure 3).

### 3.3. Phylogeny of Cypriot SARS-CoV-2 Sequences

**Figure 5:**
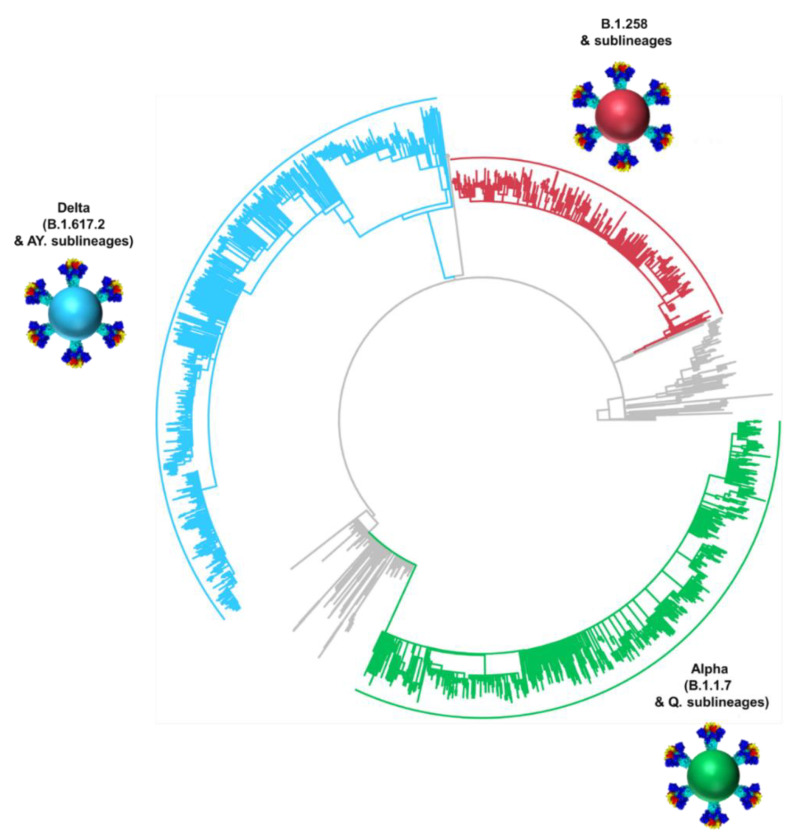
Time-scaled maximum likelihood phylogeny of all available Cypriot SARS-CoV-2 genomes. The sequences of the most prevalent lineages of the second wave (B.1.258 & sublineages), third wave (Alpha, B.1.1.7 & Q. sublineages), and fourth wave (Delta, B.1.617.2 & AY. sublineages) are highlighted in red, green and blue, respectively. Lineages that did not classify and/or cluster with B.1.258, Alpha, and Delta are denoted as “Other lineages” and indicated in gray.

The dominance of B.1.258 & sublineages, Alpha (B.1.1.7 & Q. sublineages) and Delta (B.1.617.2 & AY. sublineages) in the successive infection waves in Cyprus is reflected in their temporally spaced clustering (Figure 5), which is in line with previous findings in this study (Figure 1E,F Figure 2 and Figure 4).

### 3.4. Timed Migration Histories

**Figure 6:**
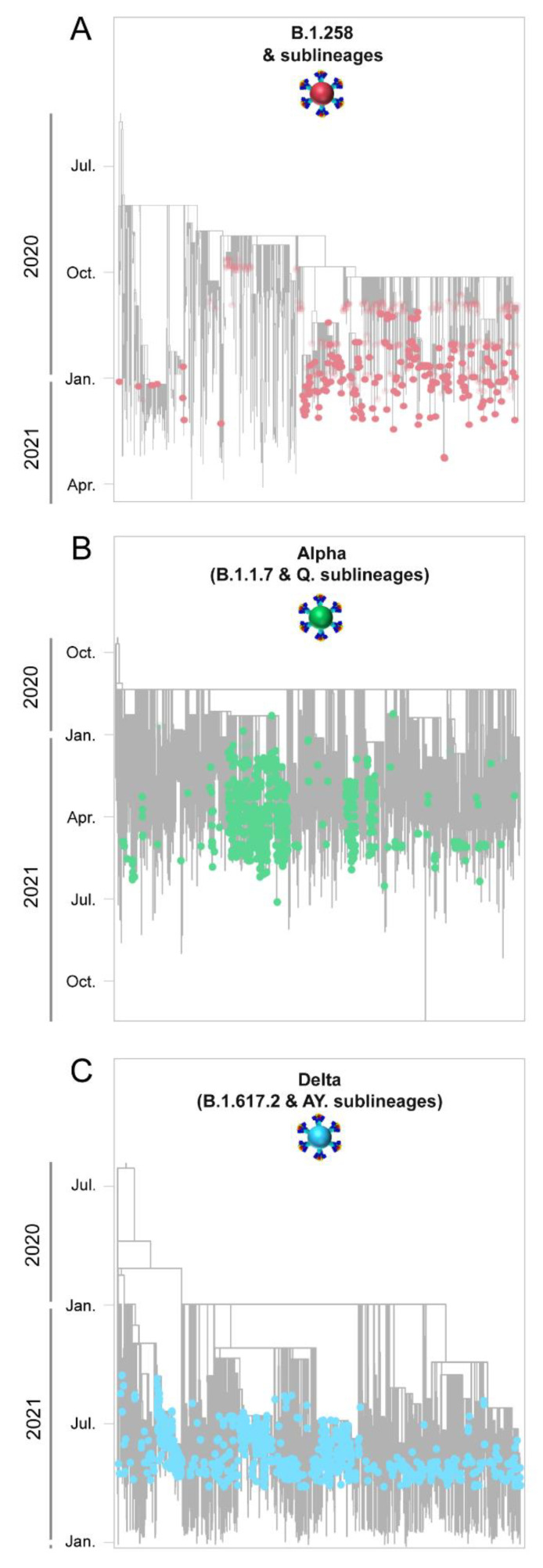
Time-scaled migration history for the datasets used for reconstructing the history of the spread of (**A**) B.1.258 & sublineages (red circles), (**B**) Alpha (B.1.1.7 & Q. sublineages, green circles), and (**C**) Delta (B.1.617.2 & AY. sublineages, blue circles). Red and green semitransparent circles represent the Cypriot B.1.258 & sublineages and Alpha (B.1.1.7 & Q. sublineages) genomes, respectively, collected during the April 2020 to January 2021 period [29]. Gray tips represent reference sequences downloaded from GISAID (accessed on 30 March 2022) [41].

**Figure 7:**
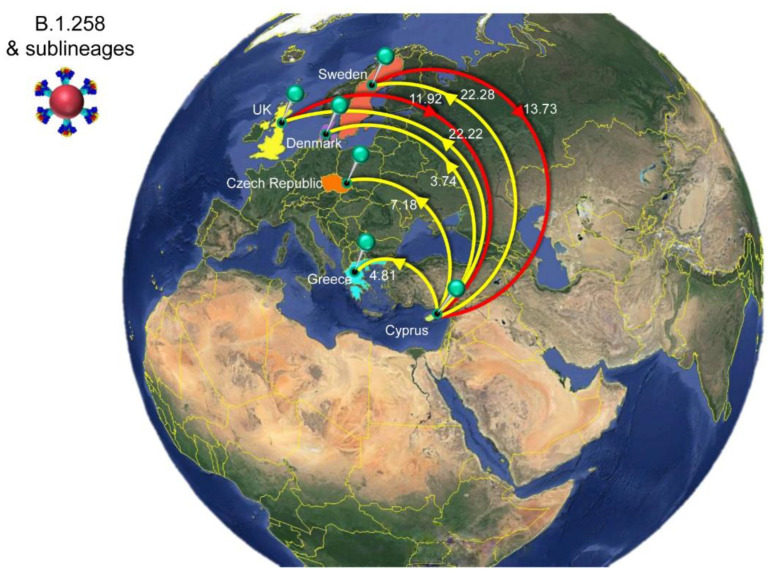
Map of SARS-CoV-2 B.1.258 & sublineage transmission between Cyprus and other countries. Geographic origins of SARS-CoV-2 B.1.258 and sublineages imported into Cyprus are shown as red lines; exports from Cyprus to other countries are shown as yellow lines. Countries acting as “sources” or “sinks” for SARS-CoV-2 B.1.258 & sublineage transmission are highlighted and labeled, and the estimated average number of migration events is indicated. Map images courtesy of Google Earth Pro 7.3.2.5776 (14 December 2015). Global view centered on Europe. 36°16′38.78″ N 36°07′29.71″ E, Eye alt 7949.12 km. US Dept. of State Geographer, DATA SIO, NOAA, U.S. Navy, NGA, GEBCO. Image Landsat/Copernicus. 2018 © Google. https://www.google.com/earth/versions/#earth-pro (accessed on 10 April 2019).

**Figure 8:**
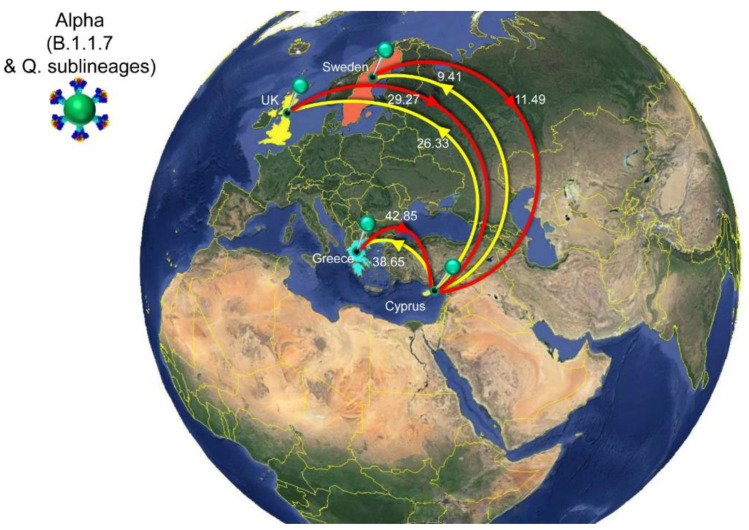
Map of SARS-CoV-2 Alpha (B.1.1.7 & Q. sublineages) transmission between Cyprus and other countries. Geographic origins of SARS-CoV-2 Alpha (B.1.1.7 & Q. sublineages) imported into Cyprus are shown as red lines; exports from Cyprus to other countries are shown as yellow lines. Countries acting as “sources” or “sinks” for SARS-CoV-2 Alpha (B.1.1.7 & Q. sublineages) transmission are highlighted and labeled, and the estimated average number of migration events is indicated. To enhance the clarity of the figure, only countries that account for 5% and above of the estimated total average number of import or export events are displayed, except if there were fewer than five countries (Table 2). Map images courtesy of Google Earth Pro 7.3.2.5776 (14 December 2015). Global view centered on Europe. 36°16′38.78″ N 36°07′29.71″ E, Eye alt 7949.12 km. US Dept. of State Geographer, DATA SIO, NOAA, U.S. Navy, NGA, GEBCO. Image Landsat/Copernicus. 2018 © Google. https://www.google.com/earth/versions/#earth-pro (accessed on 10 April 2019).

**Figure 9:**
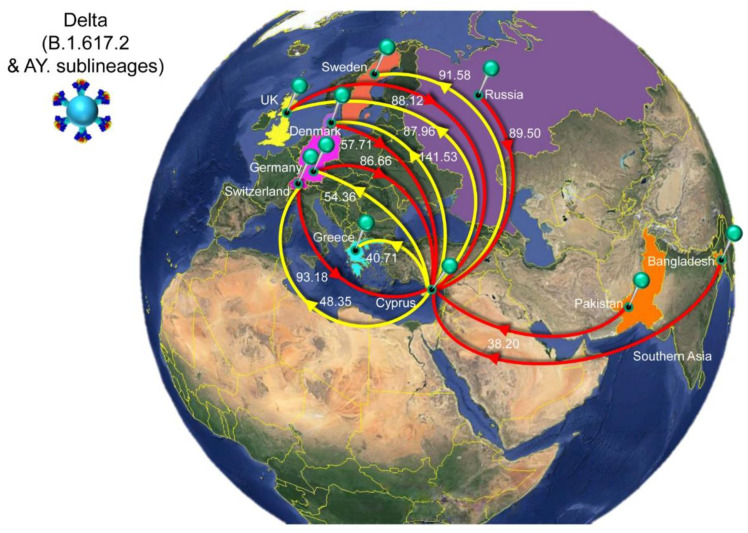
Map of SARS-CoV-2 Delta (B.1.617.2 & AY. sublineages) transmission between Cyprus and other countries. Geographic origins of SARS-CoV-2 Delta (B.1.617.2 & AY. sublineages) imported into Cyprus are shown as red lines; exports from Cyprus to other countries are shown as yellow lines. Countries and groups of countries acting as “sources” or “sinks” for SARS-CoV-2 Delta (B.1.617.2 & AY. sublineages) transmission are highlighted and labeled, and the estimated average number of migration events is indicated. The highlighted area of the countries from which migration events were from Southern Asia consists of Pakistan and Bangladesh. To enhance the clarity of the figure, only countries that account for 5% and above of the estimated total average number of importation or exportation events are displayed, except if there were fewer than five countries (Table 2). Map images courtesy of Google Earth Pro 7.3.2.5776 (14 December 2015). Center: Global view centered on Europe. 36°16′38.78″ N 36°07′29.71″ E, Eye alt 7949.12 km. US Dept. of State Geographer, DATA SIO, NOAA, U.S. Navy, NGA, GEBCO. Image Landsat/Copernicus. 2018 © Google. https://www.google.com/earth/versions/#earth-pro (accessed on 10 April 2019).

The history of spread was reconstructed for lineages that dominated the second to fourth infection waves in Cyprus (Figure 6, Figure 7, Figure 8 and Figure 9, Table 2). The locations and estimated numbers of import and export events for B.1.258 & sublineages, Alpha (B.1.1.7 & Q. sublineages), and Delta (B.1.617.2 & AY. sublineages) are shown in Figure 7, Figure 8 and Figure 9, respectively, as derived from data in Table 2.

**Figure 10:**
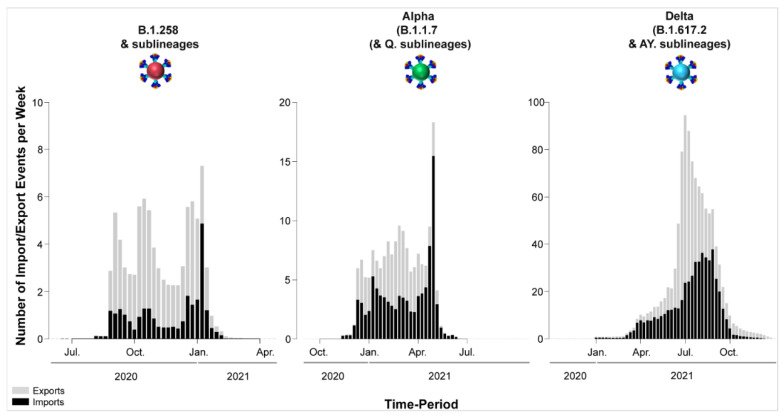
Temporal dynamics of SARS-CoV-2 export and import from and to Cyprus. The width of each column corresponds to one week, and the height corresponds to the mean of the estimated total number of migration events (to/from Cyprus) inferred to have occurred in that week. Exports are shown in gray columns; imports are shown in black columns. The y-axis represents the number of import/export events per week; the x-axis represents time.

For B.1.258 and its sublineages, the first import event to Cyprus was previously dated to 07 March 2020 (95%HPD: 17 January 2020–16 April 2020) [29]. However, no B.1.258 & sublineages were detected from June to August 2020. Hence, the likely date of the first introduction of this lineage’s variants that spread in Cyprus after the onset of the second wave in September 2020 was also estimated, which indicated that the B.1.258 & sublineages responsible for the second wave first started spreading in Cyprus on approximately 23 August 2020 (95%HPD: 6 August 2020–31 August 2020). In line with previous findings, the majority of B.1.258 and sublineage taxa cluster within a large and nearly perfectly supported clade consisting almost exclusively of genomes sampled from Cyprus [29] (Figure 6). The demographic history of this clade indicated a period of exponential growth until November 2020, after which a rather stable plateau was reached that persisted through the most recent sampling date [29]. The additional data from this study allowed for refinement of the aforementioned analysis, indicating small growth and decline periods following November 2020 until the final decline of B.1.258 lineages by February-March 2021 (Figure S1). The fact that this clade encompasses most sampled B.1.258 infections indicates that the second wave was to a large extent driven by local transmission and not by frequent importation of infections. This is reflected by the low numbers of import and export events (Table 2), with a combined maximum of less than eight per week (Figure 10). As the estimated numbers of introductions are sensitive to the sample size, these represent lower boundary estimates. In our reconstructions, we detected no more than two import events per week, except for the second week of January. Conversely, the weekly number of export events was usually higher than the number of weekly importations (Figure 10). This disparity between the weekly number of import and export events aligns with their imbalance in their overall number of import/export events: for every importation, there were 2.4 exportation events (Table 2). Although Slovenia was previously found to be a well-supported origin for import into Cyprus, this was no longer the case (i.e., Bayes factor support for a link between Slovenia and Cyprus now at >5). Instead, Sweden and the UK were identified as the only well-supported origins of B.1.258 in Cyprus, each accounting for approximately half of the import events (Figure 7, Table 2). Overall, this updated analysis of the migration patterns of B.1.258 lineages in Cyprus substantiates and refines our previous results: the UK was previously identified as a major origin location, but the additional data reveal that Sweden has also played an important role. Similarly, the UK, Czech Republic and Denmark were previously found to be well-supported destination locations for migrations out of Cyprus, and this list is now appended with Sweden and Greece (Figure 7, Table 2).

The first import event of an Alpha lineage (B.1.1.7 & Q. sublineages) was estimated to be approximately 25 November 2020 (95%HPD: 12 November 2020–08 December 2020). This date of import differs only by 6 days from our earlier estimate, which was 01 December 2020 (95%HPD: 08 November 2020–19 December 2020) [29]. Like for B.1.258, there was also a large clade with predominantly Cypriot Alpha taxa (83.5%, 457/547) (Figure 6). Despite the low support for this clade in the ML tree (SH-aLRT and UFB support values of 73.7 and 12, respectively), its presence by itself is indicative of substantial within-Cyprus spread. This is corroborated by the presence of several large well-supported subclades, as indicated by a posterior support ≥ 0.8 in analysis of this clade in BEAST v.1.10 [49] (Figure S2). In addition to this large clade, two other smaller clades with almost exclusively Cypriot taxa were noted (n = 61/72 and 78/86 taxa) and SH-aLRT/UFB support of 83.8/100 and 73.1/21, respectively. Thus, a total of 596 Cypriot Alpha variants cluster in these clades, which supports the view of substantial within-Cyprus spread of Alpha-lineage infections. The inferred total number of import/export events, however, is higher than for B.1.258 (Table 2, Figure 10), which led to higher levels of weekly import/export events compared to B.1.258 (Figure 10). Specifically, the sum of the number of import and export events was mostly higher than 5 per week, even reaching 18 per week in the beginning of May 2021 (Figure 10). The average of the estimated total number of import events was 94.09 (100%), with Greece and the UK being the primary sources of import, accounting for 42.85 (45.54%) and 29.27 (31.11%) of import events, respectively. Sweden completes the list of countries that account for > 5% of the import events into Cyprus for this lineage (Figure 8, Table 2). The observed differences between the current and previous results are most likely due to the much wider availability of infection samples due to Alpha, both in Cyprus (10 vs. 900 genomes for both studies) and elsewhere (the GISAID database [41]). Nonetheless, the UK was identified as one of the primary sources of Alpha in Cyprus in both studies. Exports of Alpha (B.1.1.7 & Q. sublineages) from Cyprus were mainly directed toward Greece and the UK. Of a total average of 74.40 (100%) export events, Greece and the UK accounted for 38.65 (51.95%) and 26.33 (35.39%) of export events, respectively. To lesser extent, Sweden was also identified as a sink of Alpha variant (B.1.1.7 & Q. sublineages) export events from Cyprus, with 9.41 (12.65%) events.

The first import event for Delta (B.1.617.2 & AY. sublineages) was estimated to be 25 December 2020 (95%HPD, 13 July 2020–21 February 2021). However, the first Delta infection was detected in April 2021. It is highly unlikely that no other Delta infection occurred between the end of December 2020 and April 2021 if Delta had already been circulating during this approximately 4-month period. Upon closer inspection, it became clear that the first introduction date was estimated as being this early because of a sparse representation of the Delta lineage diversity in the relevant section of the tree (Figure S3). The absence of taxa from elsewhere that are closely related to the relevant Cypriot taxon indicates that a long branch links the relevant Cypriot taxon to its first common ancestor with taxa from elsewhere and that the branches in the relevant clade are rather long. Because of this, the uncertainty in the location state reconstruction propagates deep in time. Therefore, the date of first introduction was estimated from a subclade with the earliest sampled Delta genomes in Cyprus, while excluding the problematic taxa shown in Figure S3. This results in an estimated first introduction date of 24 March 2021 (95%HPD: 1 November 2020–19 April 2021).

Unlike for B.1.258, Alpha and their sublineages, the Cypriot Delta (B.1.617.2 & AY. sublineages) taxa appear throughout the tree in small clusters (Figure 6), as also revealed by the markedly higher number of import and export events compared to the former two groups of lineages (B.1.258 and Alpha) (Table 2 and Figure 10). The average total number of import events for Delta in Cyprus was 521.05 (100%), with Switzerland, Russia, the UK, Germany, and Denmark being the dominant sources, accounting for 93.18 (17.88%), 89.50 (17.18%), 88.12 (16.91%), 86.66 (16.63%), and 57.71 (11.08%), respectively (Table 2). The other source of imports that accounted for >5% of the total number was Southern Asia (namely, Pakistan and Bangladesh), with 38.20 (7.33%) events (Figure 9, Table 2). Most of these locations were also identified as destinations of exports from Cyprus. Specifically, of an average total of 576.49 (100%) export events, the majority were to Denmark at 141.53 (24.55%). Other notable destinations of export (above 5% of exports) were Sweden at 91.58 (15.89%), the UK at 87.96 (15.26%), Germany at 54.36 (9.43%), Switzerland at 48.35 (8.39%), and Greece at 40.71 (7.06%) (Figure 9, Table 2). Furthermore, the four most important sources of Delta (B.1.617.2 & AY. sublineages) imports into Cyprus accounted for almost equal shares (~16–18% of all imports), which shows that this variant did not predominantly enter Cyprus through one location. Such similarity was not seen for exports.

**Table 2:** The estimated number of migration events towards and from Cyprus. Lower and upper refer to the bounds of the 95% HPD interval.

Lineage/Variant ^a^	From ^b^	To ^c^	Average ^d^	Lower ^e^	Upper ^f^
B.1.258 & sublineages	All ^g^	Cyprus	25.65	18	46
	Sweden	Cyprus	13.73	8	18
	United Kingdom	Cyprus	11.92	6	35
	Cyprus	All	60.23	39	68
	Cyprus	Sweden	22.28	18	26
	Cyprus	United Kingdom	22.22	11	27
	Cyprus	Czech Republic	7.18	0	10
	Cyprus	Greece	4.81	4	6
	Cyprus	Denmark	3.74	0	6
Alpha (B.1.1.7 & Q. sublineages)	All	Cyprus	94.09	87	100
	Greece	Cyprus	42.85	37	48
	United Kingdom	Cyprus	29.27	22	36
	Sweden	Cyprus	11.49	5	16
	Germany	Cyprus	3.46	0	9
	Bulgaria	Cyprus	3.37	3	5
	North America	Cyprus	2.07	0	6
	Africa	Cyprus	1.12	0	3
	Israel	Cyprus	0.46	0	3
	Cyprus	All	74.40	67	81
	Cyprus	Greece	38.65	34	43
	Cyprus	United Kingdom	26.33	22	31
	Cyprus	Sweden	9.41	7	13
Delta (B.1.617.2 & AY. sublineages)	All	Cyprus	521.05	487	557
	Switzerland	Cyprus	93.18	69	118
	Russia	Cyprus	89.50	66	109
	United Kingdom	Cyprus	88.12	68	103
	Germany	Cyprus	86.66	58	117
	Denmark	Cyprus	57.71	42	72
	Southern Asia	Cyprus	38.20	21	52
	Greece	Cyprus	13.04	8	18
	Italy	Cyprus	11.96	5	21
	Belgium	Cyprus	9.02	0	16
	Israel	Cyprus	7.81	4	11
	Sweden	Cyprus	6.91	0	16
	Northern America	Cyprus	4.22	0	13
	Romania	Cyprus	3.66	0	9
	Finland	Cyprus	3.25	0	14
	France	Cyprus	2.75	0	11
	Bulgaria	Cyprus	2.55	0	5
	Lithuania	Cyprus	1.82	0	4
	South-Eastern Asia	Cyprus	0.68	0	4
	Cyprus	All	576.49	524	623
	Cyprus	Denmark	141.53	118	166
	Cyprus	Sweden	91.58	78	108
	Cyprus	United Kingdom	87.96	69	107
	Cyprus	Germany	54.36	30	78
	Cyprus	Switzerland	48.35	26	70
	Cyprus	Greece	40.71	32	49
	Cyprus	Italy	20.12	11	29
	Cyprus	Netherlands	13.57	6	21
	Cyprus	Eastern Asia	13.39	8	18
	Cyprus	Israel	12.49	6	18
	Cyprus	Slovakia	11.30	6	16
	Cyprus	Bulgaria	9.54	5	14
	Cyprus	Western Europe	8.97	5	12
	Cyprus	Finland	7.27	0	16
	Cyprus	Croatia	5.47	0	11
	Cyprus	France	5.05	0	16
	Cyprus	Spain	2.42	0	8
	Cyprus	Western Asia	1.77	0	4
	Cyprus	Lithuania	0.62	0	5

^a^ Lineage/Variant refers to the Pango classification system and the WHO Greek alphabet nomenclature of naming lineages that were denoted as variants of concern (VOC) [16,17]. ^b^ “From” indicates the migration events of a country/subregion from which migration events were initiated from. Countries/subregions are as denoted by United Nations (UN) geographical subregion. ^c^ “To” indicates the migration events from a country/subregion from which migration events were directed to. Countries/subregions are as denoted by (UN) geographical subregion. ^d–f^ Represent average Markov jumps based on the lower and upper bounds of the of the 95% HPD interval migration events towards and from Cyprus. Only links supported by a base factor of at least 5 were considered. ^g^ “All” Represents the aggregation of the migration events from each country/subregion.

## 4. Discussion

In the current study, 2352 whole-genome SARS-CoV-2 sequences were analyzed and 61 SARS-CoV-2 lineages were detected in Cyprus during the period of November 2020 to October 2021. The majority of those lineages belong to three groups: B.1.258 & sublineages (303/2352, 12.88%); Alpha (B.1.1.7 & Q. sublineages) (927/2352, 39.41%); and Delta (B.1.617.2 & AY. sublineages) (977/2352, 41.54%) (Table 1).

The period of focus of the present study was November 2020 to October 2021, partly overlapping and largely extending the period of genomic surveillance of our previous work—April 2020 to January 2021 [29]; hence, there was an overlapping period from November 2020 to January 2021. The uninterrupted surveillance allowed for studying the continuous development of the SARS-CoV-2 epidemic in Cyprus, which was initially characterized by an upsurge of B.1.1 lineages (namely, B.1.1.29) (Figure 1 and Figure 2). These lineages harbor the S gene D614G mutation, which results in increased viral load and infectivity, thereby conferring a fitness advantage over lineages without this mutation [73]. Interventions including lockdown and travel restrictions contributed to mitigating the spread of SARS-CoV-2, preventing the collapse of the health and economic systems until they had adjusted to the situation [29,55,74] (Figure 1). The first full lockdown (24 March–03 May 2020), which mainly entailed a curfew and the restriction of movement without permission, was followed by a partial lockdown (04–20 May 2020). The latter mainly entailed a curfew, which was followed by the gradual lifting of travel restrictions (start of travel restrictions 04 April 2020, repatriation program 14 April 2020, gradual lift STAGE-A 09–19 June 2020 and STAGE-B 20 June 2020), with countries being categorized in accordance with their epidemiological profile at the time [74].

After restrictions were alleviated over the summer period, the SARS-CoV-2 burden began to increase again by the start of September 2020 [74] (Figure 1), which coincided with the emergence of B.1.258 and its sublineages (Figure 1 and Figure 2). The first import of B.1.258 was estimated to be 07 March 2020 (95%HPD: 17 January 2020–16 April 2020) and traces back to the detection of two B.1.258 infections as early as April-May 2020. No other B.1.258 sequences were identified during June–August 2020, which we believe is linked to the abovementioned measures, lockdowns, enhanced contact tracing, and travel restrictions that helped to mitigate the import and spread of infections (explained in detail in [29]). After these restrictions were alleviated, B.1.258 was estimated to have been reintroduced on approximately 23 August 2020 (95%HPD: 06–31 August 2020) and was first detected approximately a month later. Its prevalence continued to rise until December 2020. The B.1.258 prevalence began to decline in January 2021, and by February 2021, the second wave had ended. It is of note that the second partial lockdown was implemented 23 October 2020–9 January 2021 and was supplemented with governmental decrees that were issued depending on the epidemiological profile of the island, such as the tighter curfew during 13 November 2020–9 January 2021 for two cities in Cyprus (Limassol and Paphos) [74].

Although these interventions clearly could not completely abrogate the spread of SARS-CoV-2 infections, they are likely to have prevented an even higher burden. In turn, a more beneficial situation at the start of the subsequent complete lockdown means that the latter could have led to a quicker reduction in the SARS-CoV-2 burden. The majority of imports and exports involved Sweden and the UK, where B.1.258 was highly prevalent during this period [72,75]. The travel status of both countries was reclassified from category C (increased risk, requiring negative laboratory test results within 72 h prior to traveling, upon arrival, and 14-day isolation with testing at the end of the isolation period) to B (possibly low risk, requiring negative laboratory test results within 72 h prior to traveling from that country) in August 2020 [74]. As persons with a negative test have a small but nonzero probability of carrying SARS-CoV-2 infection [76], an imported lineage may eventually become prevalent.

The successful global spread of B.1.258 lineages is likely linked to acquisition of beneficial mutations. Two notable mutations of B.1.258 are the ΔH69/V70 deletions (Figure 3 and Figure 4). Interestingly, these deletions have recurrently emerged in other lineages, including Alpha (B.1.1.7 & Q. sublineages) [17,19]) as well as B.1.375 and B.1.1.298 [71,72]. ΔH69/V70 are located in the NTD of the S protein, a region with epitopes for neutralizing antibodies and mutational hotspots, and was even proposed to perhaps play a role in virus entry [77,78,79]. The deletions ΔH69/V70 are reported to enhance infectivity through increased spike incorporation into virions, to increase replication, and to even compensate for immune escape mutations that reduce infectivity [71,80]. Additionally, the ΔH69/V70 S gene mutations have been linked to the failure of tests used for detection of SARS-CoV-2 (S gene target failure) [81]. A second mutation, which also independently emerged in other lineages, is N439K. This site is located in the RBD of the S protein, a region of particular importance due to its involvement in binding to the host cell receptor (ACE2), and it serves a prominent target of neutralizing antibodies [82]. The N439K mutation is associated with immune evasion as well as increased affinity for ACE2 binding [80,83]. The fact that such mutations have also been detected in other lineages, some of which have become dominant, highlights the value of complementing investigations of the functional attributes of mutations with studies of their impact on epidemic spread.

The first Alpha variant infection was detected in December 2020 (Figure 1), soon after its estimated first introduction date (25 November 2020, 95%HPD: 12 November 2020–08 December 2020). This lineage was the first to be denoted as a VOC by the WHO [16] and carried a multitude of S gene mutations. Overall, the mutations characterizing the Alpha variant (B.1.1.7 & Q. sublineages) are linked to immune evasion and increased transmission/infectivity. In addition to the ΔH69/V70 deletion it carries in common with B.1.258, other mutations include the following: ΔY144, N501Y, A570D, D614G, P681H, T716I, S982A, and D1118H (Figure 3 and Figure 4). ΔY144 is located in the NTD of the S protein and was found to reduce antibody binding affinity, thereby conferring additional immune evasion potential [84]. The N501Y RBD mutation, which has also been identified in three other VOCs (Beta, Gamma and Omicron), is reported to increase ACE2 binding to the S protein RBD, enhancing infectivity [85,86]. The A570D S protein SD1 domain mutation is mainly implicated in increased infectivity [87] and may contribute to immune evasion [80,88]. On the other hand, the mutation P681H, which is adjacent to the furin cleavage site (S1/S2), has no significant impact on viral entry or the spread of the virus between cells, even though it may increase spike cleavage by furin-like proteases [88,89]. The S protein S2 subunit mutation T716I may cause a destabilization effect to favor RBD-up or open states, which is compensated for by substitutions such as D1118H, which stabilize the prefusion spike conformation [80,90]. Finally, S982A is located in the S protein S2 subunit and has been found to enhance viral entry while reducing neutralizing antibody induction [80].

The fact that the Alpha variant quickly became globally dominant after its emergence in the UK in September 2020 [91] is a testament to its competitive advantages with respect to other cocirculating lineages at the time. The highly transmissible nature of the Alpha variant is also evidenced by the fact that a complete lockdown (from 10 January 2021 to 09 May 2021, Figure 1) could not entirely control its spread, as it was able to during the first and second waves. Moreover, Greece (comprising the majority of arrivals from travelers [74,92]), the UK, and Sweden were classified as category B or C travel locations throughout the period of the partial and complete lockdowns, and phylogeographic reconstructions indicate that they were the source of 88% of the imported Alpha lineages in Cyprus (Figure 8 and Table 2) [74]. Nonetheless, the travel restriction category assignment of countries was highly dynamic [74]. The demise of the Alpha lineage in Cyprus was likely facilitated by increasing SARS-CoV-2 vaccination coverage. Indeed, vaccination began in Cyprus in December 2020/early January 2021, with priority depending on age/risk group, and by 31 May 2021, vaccination coverage had reached approximately 55% for the first dose and 35% for full vaccination in the total adult population [7,74].

Soon after the peak prevalence of Alpha variants (B.1.1.7 & Q. sublineages), Delta variants (B.1.617.2 & AY. sublineages), reported to be at least 40% more transmissible than Alpha variants [93,94], became dominant, resulting in the fourth infection wave (Figure 1 and Figure 2). The Delta variant was estimated to have been imported into Cyprus in late March (24 March 2021, 95%HPD: 1 November 2020–19 April 2021), with the first sequences being identified during April 2021 (Figure 1). Hence, only a few weeks separated the first introduction of B.1.258, Alpha and Delta and their first detection by the genomic surveillance program. In contrast to the decreasing Alpha variant prevalence near the end of the last complete lockdown, the Delta variants increased in relative importance. The number of different well-supported migration links for import to and export from Cyprus is notably higher for Delta than for B.1.258 and Alpha. We believe that to a large extent, this is linked to the combination of Delta’s increased infectivity, gradually allowing unrestricted or less restricted travel from more countries, and changes in travel intensity. At the beginning of May (10 May 2021), there were more than 40 countries in the red category, which decreased to 20 by the end of June (28 June 2021), at which point there were also 28 countries in the green category [74]. This was accompanied by an increase in the number of travelers of approximately 40,000 in April 2021 to 100,000 in May 2021 and more than 180,000 in June 2021 [92]. The travel intensity data also coincide with our results showing that Delta imports in Cyprus remained low until June and July 2021, after which there was a gradual rise in import events of this variant (Figure 10).

The fourth wave occurred during summer of 2021. Thus, climate alone, albeit impactful, cannot explain the timing of increased infection rates [95] and underscores the importance of viral genetic factors. The most commonly found Delta mutations seen in our sequences were T19R, G142D, ΔE156/F157, R158G, L452R, T478K, D614G, P681R, and D950N (Figure 3 and Figure 4). T19R, G142D, ΔE156/F157 and R158G are located within the antigenic supersite of the S protein NTD and affect immune evasion [96,97]. Furthermore, ΔE156/F157 and R158G contribute to increased infectivity [98]. The L452R and T478K S protein RBD mutations are reported to improve transmission and confer immune evasion [73,99,100]. L452R is also reported to reduce the effectiveness of antibodies induced by vaccination or natural infection [101]. P681R, adjacent to the furin cleavage site (S1/S2), improves transmission, confers partial resistance to neutralizing antibodies and enhances pathogenicity [99,102,103]. No obvious impact was reported for the S protein S2 D950N mutation, though it is hypothesized to enhance the fusogenicity of Delta spike [104].

The Delta variant is characterized by a plethora of sublineages (more than 100) denoted by the prefix “AY.” [105], and as such, the most common Delta mutations reported are based on the representation of Delta lineages in Cyprus: AY.122, 364/688 (52.91%); AY.60, 125/688, (18.17%); AY.4, 56/688 (8.14%); AY.43, 43/688 (6.25%); B.1.617.2, 41/688 (5.96%) (Table S1). Hence, if Delta in Cyprus was more commonly represented by other lineages, such as AY.4.2, which gained attention in the UK due to its slow but constant rise between July and December 2021, the S gene mutations T95I, Y145H and A222V would also be more commonly represented [106]. However, such mutations did not necessarily significantly enhance the infectivity and immune evasion of the virus in relation to the circulating lineages at the time [107].

Many of the abovementioned mutations, such as (but not limited to) ΔH69/V70, L452R, T478K, N501Y, and P681H [108], have reoccurred in a number of lineages, and their combination may confer an even more increased transmission potential to an emerging variant. Such was the case for the Omicron VOC, which incorporates over 30 S gene mutations and became the most dominant variant around the world, including Cyprus (Manuscript in preparation), soon after its detection during November 2021 [109]. As such, the Alpha, Delta and Omicron lineages are real-world examples of the principle of survival of the fittest [110].

In conclusion, this study details the ever-evolving and dynamic nature of the SARS-CoV-2 epidemic in Cyprus from November 2020 to October 2021. Most detected lineages belong to three groups that had acquired a set of S gene mutations conferring increased potential for transmission and immune evasion and are related to subsequent periods of increased SARS-CoV-2 spread in Cyprus: B.1.258 & sublineages, Alpha (B.1.1.7 & Q. sublineages), and Delta (B.1.617.2 & AY. sublineages). Reconstructions of the spatiotemporal characteristics of the epidemic consistently highlight the UK, Greece, and Sweden as important origin and destination locations of the SARS-CoV-2 spread linked to Cyprus and indicate that public health policy and interventions influenced the course of the epidemic. As such, this work confirms how genomic epidemiology can assess the efficacy of past policies and hence aid public health officials in devising disease management strategies.

## Data Availability

The sequences will be available upon publication of the manuscript on the GISAID database [41,42]. Specifically, only the 1759 sequences that were denoted as good quality under “qc.overallStatus” are available to GISAID, to avoid putative misinterpretations of mutations from data that may have derived during sequencing and assembly [35].

## Supplementary Materials

The following are available online at https://www.mdpi.com/article/10.3390/v15010108/s1, Figure S1: Population size changes in the large Cyprus-specific B.1.258 transmission lineage. Figure S2. Maximum clade credibility summary tree of the large Cypriot-specific clade in the ML tree estimated form the Alpha (B.1.1.7 & Q. sublineages) data set. Several of the internal nodes that define large subclades are well-supported (red circles indicate high posterior support of 1). Figure S3. Inset of the Delta (B.1.617.2 & AY. sublineages) MCC summary tree. Table S1: Common S-protein Mutations of B.1.258 & sublineages, Alpha (B.1.1.7 & Q. sublineages), and Delta (B.1.617.2 & AY. sublineages) that were Identified in this Dataset. Table S2: Uncommon S-protein mutations of B.1.258 & sublineages, Alpha (B.1.1.7 & Q. sublineages), and Delta (B.1.617.2 & sublineages AY.) that were identified in this dataset.

## References

[1] Lu R., Zhao X., Li J., Niu P., Yang B., Wu H., Wang W., Song H., Huang B., Zhu N. (2020). Genomic characterisation and epidemiology of 2019 novel coronavirus: Implications for virus origins and receptor binding. Lancet.

[2] Rabi F.A., Al Zoubi M.S., Al-Nasser A.D., Kasasbeh G.A., Salameh D.M. (2020). SARS-CoV-2 and coronavirus disease 2019: What we know so far. Pathogens.

[3] Demoliner M., Gularte J.S., Girardi V., Almeida P.R., de Weber M.N., Eisen A.K.A., Fleck J.D., Spilki F.R. (2021). SARS-CoV-2 and COVID-19: A perspective from environmental virology. Genet. Mol. Biol..

[4] Ritchie H., Mathieu E., Rodés-Guirao L., Appel C., Giattino C., Ortiz-Ospina E., Hasell J., Macdonald B., Beltekian D., Roser M. Coronavirus Pandemic (COVID-19). https://ourworldindata.org/coronavirus.

[5] CSSE JHU, C. for S.S. and E. (CSSE) at J.H.U. (JHU) COVID-19 Dashboard. https://gisanddata.maps.arcgis.com/apps/dashboards/bda7594740fd40299423467b48e9ecf6.

[6] Dong E., Du H., Gardner L. (2020). An interactive web-based dashboard to track COVID-19 in real time. Lancet Infect. Dis..

[7] KIOS Research and Innovation Center of Excellence (KIOS CoE) H εξάπλωση της COVID-19 στη Κύπρο (The spread of COVID-19 in Cyprus). https://covid19.ucy.ac.cy/.

[8] Pachetti M., Marini B., Benedetti F., Giudici F., Mauro E., Storici P., Masciovecchio C., Angeletti S., Ciccozzi M., Gallo R.C. (2020). Emerging SARS-CoV-2 mutation hot spots include a novel RNA-dependent-RNA polymerase variant. J. Transl. Med..

[9] Wang R., Hozumi Y., Yin C., Wei G.W. (2020). Decoding SARS-CoV-2 Transmission and Evolution and Ramifications for COVID-19 Diagnosis, Vaccine, and Medicine. J. Chem. Inf. Model..

[10] Ghadimi-Moghadam A., Haghani M., Bevelacqua J.J., Jafarzadeh A., Kaveh-Ahangar A., Mortazavi S.M.J., Ghadimi-Moghadam A., Mortazavi S.A.R. (2020). COVID-19 tragic pandemic: Concerns over unintentional “directed accelerated evolution” of novel coronavirus (SARS-CoV-2) and introducing a modified treatment method for ards. J. Biomed. Phys. Eng..

[11] Tao K., Tzou P.L., Nouhin J., Gupta R.K., de Oliveira T., Kosakovsky Pond S.L., Fera D., Shafer R.W. (2021). The biological and clinical significance of emerging SARS-CoV-2 variants. Nat. Rev. Genet..

[12] Rambaut A., Holmes E.C., O’Toole Á., Hill V., McCrone J.T., Ruis C., du Plessis L., Pybus O.G. (2020). A dynamic nomenclature proposal for SARS-CoV-2 lineages to assist genomic epidemiology. Nat. Microbiol..

[13] Cosar B., Karagulleoglu Z.Y., Unal S., Ince A.T., Uncuoglu D.B., Tuncer G., Kilinc B.R., Ozkan Y.E., Ozkoc H.C., Demir I.N. (2022). SARS-CoV-2 Mutations and their Viral Variants. Cytokine Growth Factor Rev..

[14] Telenti A., Hodcroft E.B., Robertson D.L. (2022). The Evolution and Biology of SARS-CoV-2 Variants. Cold Spring Harb. Perspect. Med..

[15] Markov P.V., Katzourakis A., Stilianakis N.I. (2022). Antigenic evolution will lead to new SARS-CoV-2 variants with unpredictable severity. Nat. Rev. Microbiol..

[16] The World Health Organization (WHO) Tracking SARS-CoV-2 Variants. https://www.who.int/en/activities/tracking-SARS-CoV-2-variants/.

[17] Choi J.Y., Smith D.M. (2021). SARS-CoV-2 variants of concern. Yonsei Med. J..

[18] Gómez C.E., Perdiguero B., Esteban M. (2021). Emerging SARS-CoV-2 variants and impact in global vaccination programs against SARS-CoV-2/COVID-19. Vaccines.

[19] Ali M., Nas F., Mu’azu L., Abdallah M. (2021). SARS-CoV-2 Variants of Concern (VOC): A Review. Clin. Res. Immunol..

[20] Giovanetti M., Fonseca V., Wilkinson E., Tegally H., San E.J., Althaus C.L., Xavier J., Nanev Slavov S., Viala V.L., Ranieri Jerônimo Lima A. (2022). Replacement of the Gamma by the Delta variant in Brazil: Impact of lineage displacement on the ongoing pandemic. Virus Evol..

[21] Tegally H., Wilkinson E., Giovanetti M., Iranzadeh A., Fonseca V., Giandhari J., Doolabh D., Pillay S., San E.J., Msomi N. (2021). Detection of a SARS-CoV-2 variant of concern in South Africa. Nature.

[22] Grubaugh N.D., Cobey S. (2021). Of variants and vaccines. Cell.

[23] Chen C., Nadeau S., Yared M., Voinov P., Xie N., Roemer C., Stadler T. (2022). CoV-Spectrum: Analysis of globally shared SARS-CoV-2 data to identify and characterize new variants. Bioinformatics.

[24] Salehi-Vaziri M., Fazlalipour M., Seyed Khorrami S.M., Azadmanesh K., Pouriayevali M.H., Jalali T., Shoja Z., Maleki A. (2022). The ins and outs of SARS-CoV-2 variants of concern (VOCs). Arch. Virol..

[25] Lino A., Cardoso M.A., Martins-Lopes P., Gonçalves H.M.R. (2022). Omicron—The new SARS-CoV-2 challenge?. Rev. Med. Virol..

[26] Viana R., Moyo S., Amoako D.G., Tegally H., Scheepers C., Althaus C.L., Anyaneji U.J., Bester P.A., Boni M.F., Chand M. (2022). Rapid epidemic expansion of the SARS-CoV-2 Omicron variant in southern Africa. Nature.

[27] Wagner A.L. What Makes a “Wave” of Disease? An Epidemiologist Explains. https://theconversation.com/what-makes-a-wave-of-disease-an-epidemiologist-explains-141573.

[28] Akande O.W., Elimian K.O., Igumbor E., Dunkwu L., Kaduru C., Olopha O.O., Ohanu D.O., Nwozor L., Agogo E., Aruna O. (2021). Epidemiological comparison of the first and second waves of the COVID-19 pandemic in Nigeria, February 2020–April 2021. BMJ Glob. Health.

[29] Chrysostomou A.C., Vrancken B., Koumbaris G., Themistokleous G., Aristokleous A., Masia C., Eleftheriou C., Iοannou C., Stylianou D.C., Ioannides M. (2021). A Comprehensive Molecular Epidemiological Analysis of SARS-CoV-2 Infection in Cyprus from April 2020 to January 2021: Evidence of a Highly Polyphyletic and Evolving Epidemic. Viruses.

[30] O’Toole Á., Scher E., Underwood A., Jackson B., Hill V., McCrone J.T., Colquhoun R., Ruis C., Abu-Dahab K., Taylor B. (2021). Assignment of epidemiological lineages in an emerging pandemic using the pangolin tool. Virus Evol..

[31] Quick J. Artic-ncov2019 Primer Schemes. https://github.com/artic-network/artic-ncov2019/blob/master/primer_schemes/nCoV-2019/V3/nCoV-2019.tsv.

[32] Li H. (2013). Aligning sequence reads, clone sequences and assembly contigs with BWA-MEM. arXiv.

[33] Chen S., Zhou Y., Chen Y., Huang T., Liao W., Xu Y., Li Z., Gu J. (2019). Gencore: An efficient tool to generate consensus reads for error suppressing and duplicate removing of NGS data. BMC Bioinform..

[34] Töpfer A. ConsensusFixer. https://github.com/cbg-ethz/consensusfixer.

[35] Aksamentov I., Roemer C., Hodcroft E., Neher R. (2021). Nextclade: Clade assignment, mutation calling and quality control for viral genomes. J. Open Source Softw..

[36] Katoh K., Standley D.M. (2013). MAFFT multiple sequence alignment software version 7: Improvements in performance and usability. Mol. Biol. Evol..

[37] Larsson A. (2014). AliView: A fast and lightweight alignment viewer and editor for large datasets. Bioinformatics.

[38] Nguyen L.T., Schmidt H.A., Von Haeseler A., Minh B.Q. (2015). IQ-TREE: A fast and effective stochastic algorithm for estimating maximum-likelihood phylogenies. Mol. Biol. Evol..

[39] Guindon S., Dufayard J.F., Lefort V., Anisimova M., Hordijk W., Gascuel O. (2010). New algorithms and methods to estimate maximum-likelihood phylogenies: Assessing the performance of PhyML 3.0. Syst. Biol..

[40] Hoang D.T., Chernomor O., Von Haeseler A., Minh B.Q., Vinh L.S. (2018). UFBoot2: Improving the ultrafast bootstrap approximation. Mol. Biol. Evol..

[41] Khare S., Gurry C., Freitas L., Schultz M.B., Bach G., Diallo A., Akite N., Ho J., Lee R.T.C., Yeo W. (2021). GISAID’s Role in Pandemic Response. China CDC Wkly..

[42] GISAID GISAID Initiative. https://www.gisaid.org/.

[43] Camacho C., Coulouris G., Avagyan V., Ma N., Papadopoulos J., Bealer K., Madden T.L. (2009). BLAST+: Architecture and applications. BMC Bioinform..

[44] To T.-H., Jung M., Lycett S., Gascuel O. (2016). Fast Dating Using Least-Squares Criteria and Algorithms. Syst. Biol..

[45] Worobey M., Pekar J., Larsen B.B., Nelson M.I., Hill V., Joy J.B., Rambaut A., Suchard M.A., Wertheim J.O., Lemey P. (2020). The emergence of SARS-CoV-2 in Europe and North America. Science.

[46] Lemey P., Rambaut A., Bedford T., Faria N., Bielejec F., Baele G., Russell C.A., Smith D.J., Pybus O.G., Brockmann D. (2014). Unifying Viral Genetics and Human Transportation Data to Predict the Global Transmission Dynamics of Human Influenza H3N2. PLoS Pathog..

[47] Lemey P., Rambaut A., Drummond A.J., Suchard M.A. (2009). Bayesian phylogeography finds its roots. PLoS Comput. Biol..

[48] Edwards C.J., Suchard M.A., Lemey P., Welch J.J., Barnes I., Fulton T.L., Barnett R., O’Connell T.C., Coxon P., Monaghan N. (2011). Ancient hybridization and an irish origin for the modern polar bear matriline. Curr. Biol..

[49] Suchard M.A., Lemey P., Baele G., Ayres D.L., Drummond A.J., Rambaut A. (2018). Bayesian phylogenetic and phylodynamic data integration using BEAST 1.10. Virus Evol..

[50] Minin V.N., Suchard M.A. (2008). Fast, accurate and simulation-free stochastic mapping. Philos. Trans. R. Soc. B Biol. Sci..

[51] Hasegawa M., Kishino H., Yano T. (1985). Dating of the human-ape splitting by a molecular clock of mitochondrial DNA. J. Mol. Evol..

[52] Uzzell T., Corbin K.W. (1971). Fitting Discrete Probability Distributions to Evolutionary Events. Science.

[53] Gill M.S., Lemey P., Faria N.R., Rambaut A., Shapiro B., Suchard M.A. (2013). Improving bayesian population dynamics inference: A coalescent-based model for multiple loci. Mol. Biol. Evol..

[54] Centers for Disease Control and Prevention (CDC) Calculating SARS-CoV-2 Laboratory Test Percent Positivity: CDC Methods and Considerations for Comparisons and Interpretation. https://www.cdc.gov/coronavirus/2019-ncov/lab/resources/calculating-percent-positivity.html.

[55] Press and Information Office Aνακοινωθέντα (Press Releases)-Aνακοίνωση του Υπουργείου Υγείας για νέα περιστατικά της νόσου COVID-19 (Announcement of the Ministry of Health of New COVID-19 Incidents. https://www.pio.gov.cy/ανακοινωθέντα/?keyword=Aνακοίνωση+του+Υπουργείου+Υγείας+για+νέα+περιστατικά+της+νόσου+COVID-19&startdate=&enddate=&category=&submitbtn=Aναζήτηση.

[56] Bateman A., Martin M.J., O’Donovan C., Magrane M., Alpi E., Antunes R., Bely B., Bingley M., Bonilla C., Britto R. (2017). UniProt: The universal protein knowledgebase. Nucleic Acids Res..

[57] Liu L., Wang P., Nair M.S., Yu J., Rapp M., Wang Q., Luo Y., Chan J.F.W., Sahi V., Figueroa A. (2020). Potent neutralizing antibodies against multiple epitopes on SARS-CoV-2 spike. Nature.

[58] Wang Q., Ma J., Acevedo A. (2021). High-Potency Polypeptide-based Interference for Coronavirus Spike Glycoproteins. BioRxiv.

[59] Khelashvili G., Plante A., Doktorova M., Weinstein H. (2021). Ca(2+)-dependent mechanism of membrane insertion and destabilization by the SARS-CoV-2 fusion peptide. Biophys. J..

[60] Wang P., Nair M.S., Liu L., Iketani S., Luo Y., Guo Y., Wang M., Yu J., Zhang B., Kwong P.D. (2021). Antibody Resistance of SARS-CoV-2 Variants, B.1.351 and B.1.1.7. Nature.

[61] Kim S., Lee J.H., Lee S., Shim S., Nguyen T.T., Hwang J., Kim H., Choi Y.O., Hong J., Bae S. (2020). The progression of sars coronavirus 2 (SARS-CoV-2): Mutation in the receptor binding domain of spike gene. Immune Netw..

[62] Mittal A., Manjunath K., Ranjan R.K., Kaushik S., Kumar S., Verma V. (2020). COVID-19 pandemic: Insights into structure, function, and hACE2 receptor recognition by SARS-CoV-2. PLoS Pathog..

[63] Huang Y., Yang C., Xu X.F., Xu W., Liu S.W. (2020). Structural and functional properties of SARS-CoV-2 spike protein: Potential antivirus drug development for COVID-19. Acta Pharmacol. Sin..

[64] Xia X. (2021). Domains and Functions of Spike Protein in SARS-CoV-2 in the Context of Vaccine Design. Viruses.

[65] Gobeil S.M.C., Janowska K., McDowell S., Mansouri K., Parks R., Manne K., Stalls V., Kopp M.F., Henderson R., Edwards R.J. (2021). D614G Mutation Alters SARS-CoV-2 Spike Conformation and Enhances Protease Cleavage at the S1/S2 Junction. Cell Rep..

[66] Sasaki M., Uemura K., Sato A., Toba S., Sanaki T., Maenaka K., Hall W.W., Orba Y., Sawa H. (2021). SARS-CoV-2 variants with mutations at the S1/ S2 cleavage site are generated in vitro during propagation in TMPRSS2-deficient cells. PLoS Pathog..

[67] Kapoor K., Chen T., Tajkhorshid E. (2022). Posttranslational modifications optimize the ability of SARS-CoV-2 spike for effective interaction with host cell receptors. Proc. Natl. Acad. Sci. USA.

[68] De Marco C., Veneziano C., Massacci A., Pallocca M., Marascio N., Quirino A., Barreca G.S., Giancotti A., Gallo L., Lamberti A.G. (2022). Dynamics of Viral Infection and Evolution of SARS-CoV-2 Variants in the Calabria Area of Southern Italy. Front. Microbiol..

[69] de Hoffer A., Vatani S., Cot C., Cacciapaglia G., Chiusano M.L., Cimarelli A., Conventi F., Giannini A., Hohenegger S., Sannino F. (2022). Variant-driven early warning via unsupervised machine learning analysis of spike protein mutations for COVID-19. Sci. Rep..

[70] Mathias M., do Nascimento G.M., Nooruzzaman M., Yuan F., Chen C., Caserta L.C., Miller A.D., Whittaker G.R., Fang Y., Diel D.G. (2022). The Omicron Variant BA.1.1 Presents a Lower Pathogenicity than B.1 D614G and Delta Variants in a Feline Model of SARS-CoV-2 Infection. J. Virol..

[71] Meng B., Kemp S.A., Papa G., Datir R., Ferreira I.A.T.M., Marelli S., Harvey W.T., Lytras S., Mohamed A., Gallo G. (2021). Recurrent emergence of SARS-CoV-2 spike deletion H69/V70 and its role in the Alpha variant B.1.1.7. Cell Rep..

[72] Brejová B., Boršová K., Hodorová V., Čabanová V., Reizigová L., Paul E.D., Čekan P., Klempa B., Nosek J., Vinař T. (2021). A SARS-CoV-2 mutant from B.1.258 lineage with ∆H69/∆V70 deletion in the Spike protein circulating in Central Europe in the fall 2020. Virus Genes.

[73] Thakur V., Bhola S., Thakur P., Patel S.K.S., Kulshrestha S., Ratho R.K., Kumar P. (2022). Waves and variants of SARS-CoV-2: Understanding the causes and effect of the COVID-19 catastrophe. Infection.

[74] Republic of Cyprus Ministry of Health New Coronavirus Disease (COVID-19). https://www.pio.gov.cy/coronavirus/eng/categories/important-announcements.

[75] Wu J., Zhang L., Zhang Y., Wang H., Ding R., Nie J., Li Q., Liu S., Yu Y., Yang X. (2021). The Antigenicity of Epidemic SARS-CoV-2 Variants in the United Kingdom. Front. Immunol..

[76] Tande A.J., Binnicker M.J., Ting H.H., Del Rio C., Jalil L., Brawner M., Carter P.W., Toomey K., Shah N.D., Berbari E.F. (2021). SARS-CoV-2 Testing Before International Airline Travel, December 2020 to May 2021. Mayo Clin. Proc..

[77] Kubik S., Arrigo N., Bonet J., Xu Z. (2021). Mutational Hotspot in the SARS-CoV-2 Spike Protein N-Terminal Domain Conferring Immune Escape Potential. Viruses.

[78] Klinakis A., Cournia Z., Rampias T. (2021). N-terminal domain mutations of the spike protein are structurally implicated in epitope recognition in emerging SARS-CoV-2 strains. Comput. Struct. Biotechnol. J..

[79] Awasthi M., Gulati S., Sarkar D.P., Tiwari S., Kateriya S., Ranjan P., Verma S.K. (2020). The Sialoside-Binding Pocket of SARS-CoV-2 Spike Glycoprotein Structurally Resembles MERS-CoV. Viruses.

[80] Peng Q., Zhou R., Liu N., Wang H., Xu H., Zhao M., Yang D., Au K.-K., Huang H., Liu L. (2022). Naturally occurring spike mutations influence the infectivity and immunogenicity of SARS-CoV-2. Cell. Mol. Immunol..

[81] McMillen T., Jani K., Robilotti E.V., Kamboj M., Babady N.E. (2022). The spike gene target failure (SGTF) genomic signature is highly accurate for the identification of Alpha and Omicron SARS-CoV-2 variants. Sci. Rep..

[82] Torbati E., Krause K.L., Ussher J.E. (2021). The Immune Response to SARS-CoV-2 and Variants of Concern. Viruses.

[83] Thomson E.C., Rosen L.E., Shepherd J.G., Spreafico R., da Silva Filipe A., Wojcechowskyj J.A., Davis C., Piccoli L., Pascall D.J., Dillen J. (2021). Circulating SARS-CoV-2 spike N439K variants maintain fitness while evading antibody-mediated immunity. Cell.

[84] McCarthy K.R., Rennick L.J., Nambulli S., Robinson-McCarthy L.R., Bain W.G., Haidar G., Duprex W.P. (2021). Recurrent deletions in the SARS-CoV-2 spike glycoprotein drive antibody escape. Science.

[85] Lee C., Mangalaganesh S., Wilson L.O.W., Kuiper M.J., Drew T.W., Vasan S.S. (2022). Tracking Co-Occurrence of N501Y, P681R, and Other Key Mutations in SARS-CoV-2 Spike for Surveillance. Zoonotic Dis..

[86] McCallum M., Czudnochowski N., Rosen L.E., Zepeda S.K., Bowen J.E., Walls A.C., Hauser K., Joshi A., Stewart C., Dillen J.R. (2022). Structural basis of SARS-CoV-2 Omicron immune evasion and receptor engagement. Science.

[87] Yang T.-J., Yu P.-Y., Chang Y.-C., Liang K.-H., Tso H.-C., Ho M.-R., Chen W.-Y., Lin H.-T., Wu H.-C., Hsu S.-T.D. (2021). Effect of SARS-CoV-2 B.1.1.7 mutations on spike protein structure and function. Nat. Struct. Mol. Biol..

[88] Eslami S., Glassy M.C., Ghafouri-Fard S. (2022). A comprehensive overview of identified mutations in SARS CoV-2 spike glycoprotein among Iranian patients. Gene.

[89] Lubinski B., Fernandes M.H.V., Frazier L., Tang T., Daniel S., Diel D.G., Jaimes J.A., Whittaker G.R. (2022). Functional evaluation of the P681H mutation on the proteolytic activation of the SARS-CoV-2 variant B.1.1.7 (Alpha) spike. iScience.

[90] Gobeil S.M.-C., Janowska K., McDowell S., Mansouri K., Parks R., Stalls V., Kopp M.F., Manne K., Li D., Wiehe K. (2022). Effect of natural mutations of SARS-CoV-2 on spike structure, conformation, and antigenicity. Science.

[91] Domingo P., de Benito N. (2021). Alpha variant SARS-CoV-2 infection: How it all starts. eBioMedicine.

[92] Cyprus Statistical Service (2021). Arrivals of Tourists by Country of Usual Residence. https://www.cystat.gov.cy/en/KeyFiguresList?s=51&fbclid=IwAR0mThPdhjg-Uj64Q2kAW7ibreEZgx1i4PftJZ_orJimRXU1AOKpbUXExB0.

[93] Callaway E. (2021). The mutation that helps Delta spread like wildfire. Nature.

[94] von Wintersdorff C.J.H., Dingemans J., van Alphen L.B., Wolffs P.F.G., van der Veer B.M.J.W., Hoebe C.J.P.A., Savelkoul P.H.M. (2022). Infections with the SARS-CoV-2 Delta variant exhibit fourfold increased viral loads in the upper airways compared to Alpha or non-variants of concern. Sci. Rep..

[95] Liu X., Huang J., Li C., Zhao Y., Wang D., Huang Z., Yang K. (2021). The role of seasonality in the spread of COVID-19 pandemic. Environ. Res..

[96] McCallum M., Walls A.C., Sprouse K.R., Bowen J.E., Rosen L.E., Dang H.V., De Marco A., Franko N., Tilles S.W., Logue J. (2021). Molecular basis of immune evasion by the Delta and Kappa SARS-CoV-2 variants. Science.

[97] Singh P., Sharma K., Singh P., Bhargava A., Negi S.S., Sharma P., Bhise M., Tripathi M.K., Jindal A., Nagarkar N.M. (2022). Genomic characterization unravelling the causative role of SARS-CoV-2 Delta variant of lineage B.1.617.2 in 2nd wave of COVID-19 pandemic in Chhattisgarh, India. Microb. Pathog..

[98] Mishra T., Dalavi R., Joshi G., Kumar A., Pandey P., Shukla S., Mishra R.K., Chande A. (2022). SARS-CoV-2 spike E156G/Δ157-158 mutations contribute to increased infectivity and immune escape. Life Sci. Alliance.

[99] Dhawan M., Sharma A., Priyanka, Thakur N., Rajkhowa T.K., Choudhary O.P. (2022). Delta variant (B.1.617.2) of SARS-CoV-2: Mutations, impact, challenges and possible solutions. Hum. Vaccin. Immunother..

[100] Bhattacharya M., Chatterjee S., Sharma A.R., Lee S.S., Chakraborty C. (2022). Delta variant (B. 1.617. 2) of SARS-CoV-2: Current understanding of infection, transmission, immune escape, and mutational landscape. Folia Microbiol..

[101] Wilhelm A., Toptan T., Pallas C., Wolf T., Goetsch U., Gottschalk R., Vehreschild M.J.G.T., Ciesek S., Widera M. (2021). Antibody-Mediated Neutralization of Authentic SARS-CoV-2 B.1.617 Variants Harboring L452R and T478K/E484Q. Viruses.

[102] Liu Y., Liu J., Johnson B.A., Xia H., Ku Z., Schindewolf C., Widen S.G., An Z., Weaver S.C., Menachery V.D. (2022). Delta spike P681R mutation enhances SARS-CoV-2 fitness over Alpha variant. Cell Rep..

[103] Saito A., Irie T., Suzuki R., Maemura T., Nasser H., Uriu K., Kosugi Y., Shirakawa K., Sadamasu K., Kimura I. (2022). Enhanced fusogenicity and pathogenicity of SARS-CoV-2 Delta P681R mutation. Nature.

[104] He X., He C., Hong W., Zhang K., Wei X. (2021). The challenges of COVID-19 Delta variant: Prevention and vaccine development. MedComm.

[105] Focosi D., Maggi F., Mcconnell S., Casadevall A. (2022). Spike mutations in SARS-CoV-2 AY sublineages of the Delta variant of concern: Implications for the future of the pandemic. Future Microbiol..

[106] Saunders N., Planas D., Bolland W.H., Rodriguez C., Fourati S., Buchrieser J., Planchais C., Prot M., Staropoli I., Guivel-Benhassine F. (2022). Fusogenicity and neutralization sensitivity of the SARS-CoV-2 Delta sublineage AY.4.2. eBioMedicine.

[107] Arora P., Kempf A., Nehlmeier I., Graichen L., Winkler M.S., Lier M., Schulz S., Jäck H.-M., Pöhlmann S., Hoffmann M. (2022). No evidence for increased cell entry or antibody evasion by Delta sublineage AY.4.2. Cell. Mol. Immunol..

[108] Chugh A., Khurana N., Verma K., Sehgal I., Rolta R., Vats P., Phartyal R., Salaria D., Kaushik N., Choi E.H. (2022). Changing Dynamics of SARS-CoV-2: A Global Challenge. Appl. Sci..

[109] Fan Y., Li X., Zhang L., Wan S., Zhang L., Zhou F. (2022). SARS-CoV-2 Omicron variant: Recent progress and future perspectives. Signal Transduct. Target. Ther..

[110] Darwin C. (2004). On the Origin of Species, 1859.

