# Supplementary material for: Genomic Epidemiology of the SARS-CoV-2 Epidemic in Cyprus from November 2020 to October 2021: The Passage of Waves of Alpha and Delta Variants of Concern
Source: Viruses. 2022 Dec 30;15(1):108. doi: 10.3390/v15010108 (PMC9862594; doi:10.3390/v15010108)
Supplement: Supplementary file 1 [file viruses-15-00108-s001.zip › viruses-2120792-SI.pdf]

Table S1. Common S-protein Mutations of B.1.258 & sublineages, Alpha (B.1.1.7 & Q. sublineages), and Delta (B.1.617.2 & AY. sublineages) that were Identified in this Dataset[a].

Total Sequences[b]	Lineage[c]	Mutations and Number of Sequences Identified for Each Mutation										
258	B.1.258	ΔH69	ΔV70	N439K	D614G							
		258	258	258	258							
3	B.1.258.17	ΔH69	ΔV70	L189F	N439K	D614G	V772I					
		3	3	3	3	3	3					
2	B.1.258.22	A67V	ΔH69	ΔV70	N439K	D614G						
		2	2	2	2	2						
690	B.1.1.7	ΔH69	ΔV70	ΔY144	N501Y	A570D	D614G	P681H	T716I	S982A	D1118H	
		690	690	689	686	679	690	676	689	690	690	
1	Q.8	ΔH69	ΔV70	ΔY144	N501Y	A570D	D614G	P681H	T716I	S982A	D1118H	
		1	1	1	1	1	1	1	1	1	1	
41	B.1.617.2	T19R	G142D[d]	ΔE156[e]	ΔF157[e]	R158G[e]	L452R	T478K	D614G	P681R	D950N	
		41	18	41	41	41	41	41	41	40	41	
1	AY.1	T19R	W258L	K417N	T478K	D614G	P681R	D950N				
		1	1	1	1	1	1	1				
56	AY.4	T19R	T95I	G142D[d]	ΔE156	ΔF157	R158G	L452R	T478K	D614G	P681R	D950N
		56	54	20	54	54	54	56	56	56	50	56
2	AY.4.2	T19R	T95I	Y145H	ΔE156	ΔF157	R158G	A222V	L452R	T478K	D614G	P681R
		2	2	2	2	2	2	2	2	2	2	2
		D950N										
		2										
1	AY.4.3	T19R	P26S	T95I	ΔE156	ΔF157	R158G	L452R	T478K	D614G	P681R	D950N
		1	1	1	1	1	1	1	1	1	1	1
3	AY.4.4	L5F	T19R	T95I[d]	G142D[d]	ΔE156	ΔF157	R158G	L452R	T478K	D614G	P681R[d]
		3	3	2	1	3	3	3	3	3	3	2
		S929T[d]	D950N									
		2	3									
1	AY.4.5	T19R	T95I	G142D	ΔE156	ΔF157	R158G	L452R	T478K	D614G	P681R	D950N
		1	1	1	1	1	1	1	1	1	1	1
		D1259Y										
		1										
1	AY.5	T19R	ΔE156	ΔF157	R158G	L452R	T478K	D614G	P681R	D950N		
		1	1	1	1	1	1	1	1	1		
2	AY.6	T19R	G142D[d]	ΔE156	ΔF157	R158G	L452R	T478K	D614G	P681R	D950N	
		2	1	2	2	2	2	2	2	2	2	
1	AY.7	T19R	G142D	ΔE156	ΔF157	R158G	L452R	T478K	D614G	P681R	D950N	
		1	1	1	1	1	1	1	1	1	1	
1	AY.7.2	T19R	G142D[f]	ΔE156	ΔF157	R158G	L452R	T478K	D614G	D950N		
		1	1	1	1	1	1	1	1	1		
6	AY.9	T19R	G142D[d]	ΔE156	ΔF157	R158G	A222V	L452R	T478K	D614G	P681R	D950N
		6	3	6	6	6	6	6	6	6	5	6
5	AY.9.2	T19R	G142D[d]	ΔE156	ΔF157	R158G	A222V	L452R	T478K	D614G	P681R	D950N
		5	3	5	5	5	5	5	5	5	4	5
1	AY.23	T19R	G142D	ΔE156	ΔF157	R158G	L452R	T478K	D614G	D950N	V1264L	

		1	1	1	1	1	1	1	1	1	1	
1	AY.25.1	T19R	ΔE156	ΔF157	R158G	L452R	T478K	D614G	P681R	D950N		
		1	1	1	1	1	1	1	1	1		
1	AY.34.1	T19R	T95I	ΔE156	ΔF157	R158G	L452R	T478K	D614G	Q677H	P681R	D950N
		1	1	1	1	1	1	1	1	1	1	1
		S:L1265F										
		1										
1	AY.36	T19R	T95I	ΔE156	ΔF157	R158G	L452R	T478K	D614G	P681R	D950N	V1104L
		1	1	1	1	1	1	1	1	1	1	1
1	AY.42	T19R	T95I	L452R	T478K	D614G	P681R	D950N				
		1	1	1	1	1	1	1				
43	AY.43	T19R	G142D[d]	ΔE156	ΔF157	R158G	L452R	T478K	D614G	P681R	D950N	
		43	13	43	43	43	43	43	43	40	43	
2	AY.44	T19R	ΔE156	ΔF157	R158G	L452R	T478K	D614G	P681R	D950N		
		2	2	2	2	2	2	2	2	2		
1	AY.46	T19R	G142D	ΔE156	ΔF157	R158G	L452R	T478K	D614G	P681R	D950N	
		1	1	1	1	1	1	1	1	1	1	
3	AY.46.6	T19R	E96Q[f]	ΔE156	ΔF157	R158G	A222S[f]	L452R	T478K	D614G	P681R	D950N
		3	3	3	3	3	3	3	3	3	3	3
125	AY.60	T19R	G142D[f]	ΔE156	ΔF157	R158G	A222V	L452R	T478K	D614G	P681R	D950N
		125	114	125	125	125	125	125	125	125	116	125
2	AY.98	T19R	G142D[d]	ΔE156	ΔF157	R158G	L452R	T478K	D614G	P681R	D950N	
		2	1	2	2	2	2	2	2	2	2	
8	AY.98.1	T19R	ΔE156	ΔF157	R158G	P251L	L452R	T478K	D614G	P681R	D950N	
		8	8	8	8	8	8	8	8	8	7	
1	AY.103	T19R	G142D	ΔE156	ΔF157	R158G	L452R	T478K	D614G	P681R	D950N	
		1	1	1	1	1	1	1	1	1	1	
3	AY.120	T19R	T95I	G142D[d]	ΔE156	ΔF157	R158G	L452R	T478K	D614G	P681R[d]	D950N
		3	3	2	3	3	3	3	3	3	2	3
364	AY.122	T19R	G142D	ΔE156	ΔF157	R158G	L452R	T478K	D614G	P681R	D950N	
		364	287	362	362	362	364	364	364	326	361	
3	AY.125	T19R	T95I	ΔE156	ΔF157	R158G	L452R	T478K	D614G	P681R	D950N	
		3	3	3	3	3	3	3	3	3	3	
3	AY.126	T19R	T95I	ΔE156	ΔF157	R158G	L452R	T478K	D614G	P681R[d]	I850L	D950N
		3	3	3	3	3	3	3	3	2	3	3
4	AY.128	T19R	T95I	ΔE156	ΔF157	R158G	A435S[f]	L452R	T478K	D614G	P681R	D950N
		4	3	4	4	4	4	4	4	4	4	4

[a]The most prevalent mutations shown in Table S1 were determined in accordance to the most prevalent mutations of these lineages in this dataset, and as denoted by the CoV-Spectrum [1](https://cov-spectrum.org/)(date accessed 30 May 2022) and Outbreak.info (https://outbreak.info/)(date accessed 30 May 2022) websites[2].
[b]Total number of sequences that were identified for each lineage in this dataset.
[c]For lineages that were identified in lower frequency in this dataset, the CoV-Spectrum [1](https://cov-spectrum.org/)(date accessed 30 May 2022) and Outbreak.info (https://outbreak.info/)(date accessed 30 May 2022) websites[2] were used to indicate the expected common mutations and deletions

[d] Mutations that are listed as common (above 75% prevalence) by CoV-Spectrum [1](https://cov-spectrum.org/)(date accessed 30 May 2022) and Outbreak.info (https://outbreak.info/)(date accessed 30 May 2022) websites[2], for a specific lineage but were found in lower frequencies (below 75%) in this dataset.

[e] The mutations and deletions ΔE156, ΔF157 and R158G are sometimes listed as E156G, ΔF157 and ΔR158 https://covariants.org/variants/21A.Delta (date accessed 31 May 2022) [3,4].

[f] Mutations that are listed as below 75% prevalence in CoV-Spectrum [1](https://cov-spectrum.org/)(date accessed 30 May 2022) and Outbreak.info (https://outbreak.info/)(date accessed 30 May 2022) websites[2], but were found in higher frequencies (above 75%) in this dataset for specific lineages.

Table S2. Uncommon S-protein mutations of B.1.258 & sublineages, Alpha (B.1.1.7 & Q. sublineages), and Delta (B.1.617.2 & sublineages AY.) that were identified in this dataset[a]

Total Sequences[b]	Lineage	Mutations and Number of Sequences Identified for Each Mutation										
258	B.1.258	S12F	G75V	T95I	ΔD138	ΔP139	ΔF140	ΔL141	ΔG142	ΔV143	ΔY144	ΔY145
		1	1	4	1	1	1	1	1	1	2	1
		R158G	M177I	L179F	G199V	S221L	S256L	W258L	E281K	T299I	F490S	K529N
		1	2	1	1	1	10	1	30	12	1	1
		T547I	E654Q	H655Y	T678I	N703Y	P715S	P793S	D808N	S813I	A845V	A846V
		1	5	1	1	5	1	1	1	1	1	1
		A893S	D936Y	S968A	K1045R	A1078V	W1102L	D1118Y	D1163Y	V1176F	L1203F	
		4	1	4	1	4	4	1	4	5	2	
690	B.1.1.7	L5F	L8F	S13I	T20I	T22I	P26L	ΔV42	ΔF43	ΔR44	ΔS45	ΔS46
		6	1	1	1	3	1	1	1	1	1	1
		A67V	V90F	S98F	D138H	ΔG142	Y144V	N148T	F157L	S162I	Q173K	E180K
		1	1	1	1	1	1	1	93	1	1	1
		G181V	V213L	D215Y	L216F	G219D	G261V	P272L	V308L	P330S	V362F	V367F
		1	2	1	1	1	1	4	1	1	1	1
		L390F	E309A	G413R	S494P	G496S	A520S	L552F	T553I	D568N	T572I	E583D
		1	7	9	1	1	1	1	1	1	1	3
		T618I	A626V	F643L	A653V	E654Q	Q677H	A684V	V687I	A694S	S704L	V705F
		1	1	2	1	1	1	2	2	8	1	1
		E748Q	L752F	T778I	A783S	P812S	D830Y	A831V	M869I	A871S	A879V	A893V
		4	1	1	1	1	1	58	1	1	1	1
		A924S	A924V	A930V	L938F	S939F	M1050I	V1065A	A1078V	D1084E	A1087S	R1091H
		1	1	1	1	1	2	1	1	6	7	1
		D1163H	G1167V	E1195Q	Q1201H	L1203F	G1219C	G1219V	M1229I	M1229T	V1230L	C1243F
		1	5	1	1	1	3	6	2	1	7	1
41	B.1.617.2	T95I	D111N	Y145H	A222V	D253G	T572I	Q613H	Q677H	N703S	V772I	M1237I
		24	1	1	6	1	1	1	4	2	1	1
		K1255I	V1264L									
		1	4									
56	AY.4	A67V	Q183R	V213L	G446V	L455F	N532Y	K558N	H655Y	T859I	A892V	A1087S
		1	4	1	1	1	1	1	3	1	1	1
		D1163Y	P1263L	V1264L								

		1	2	1								
1	AY.4.5	R273S										
		1										
2	AY.6	T22I										
		1										
6	AY.9	S46A	G181L	T1117I								
		1	1	1								
1	AY.42	T719I										
		1										
43	AY.43	L5F	S112L	G181V	V213A	N354K	V401L	A570S	T572I	Q613H	A623T	R683L
		1	1	1	3	3	1	6	1	1	3	6
		A871V	A942S	T1116N	G1124V	S1170F	I1232T	D1259Y				
		4	1	1	2	2	1	1				
1	AY.46	Q613H										
		1										
125	AY.60	D80Y	T95I	E96Y	N125D	G142Y	V362F	V401L	T547I	S813I	D830Y	A1078S
		5	2	3	1	2	2	1	1	2	1	1
		H1101Y	C1236F	K1255I								
		1	1	1								
2	AY.98	K854N										
		1										
8	AY.98.1	T547I										
		1										
3	AY.120	P1263L										
		1										
364	AY.122	L5F	Q14H	V16F	H49Y	L54F	D80Y	W152L	M153I	G181V	L189F	I231T
		1	1	3	1	4	1	1	1	5	1	1
		Y449S	W258L	A260S	Y266C	T274I	Y279F	L293F	V308L	E309Q	R346G	V367I
		1	1	1	1	1	1	1	4	2	2	1
		A372V	T376N	I402L	V483F	T547I	K558N	A570S	D574Y	L582R	Q613H	ΔI624
		1	1	1	1	1	1	4	1	1	1	1
		ΔH625	ΔA626	ΔD627	ΔQ628	ΔL629	ΔT630	ΔP631	ΔT632	ΔW633	ΔR634	ΔV635
		1	1	1	1	1	1	1	1	1	1	1

		Y636N	A647S	A653V	Q677H	S680T	A684V	A701V	R765C	G769V	I805V	P812S
		1	1	3	1	1	1	1	2	2	1	1
		S929I	S937T	S940F	A1070V	A1078S	V1104L	G1124V	D1153Y	V1176F	V1177L	Y1215H
		1	1	1	1	1	1	1	1	2	7	1
		G1219V	M1229I	C1243F	K1245N	C1250F	C1250Y					
		1	2	1	1	1	1					
3	AY.125	G142D										
		1										
3	AY.126	G142D	E281Q									
		1	1									

[a]The uncommon mutations shown in Table S2 were determined in accordance to the uncommon mutations of the lineages in this dataset, and as denoted by the CoV-Spectrum [1](https://cov-spectrum.org/)(date accessed 30 May 2022) and Outbreak.info (https://outbreak.info/)(date accessed 30 May 2022) websites[2].

[b]Total number of sequences that were identified for each lineage in this dataset.

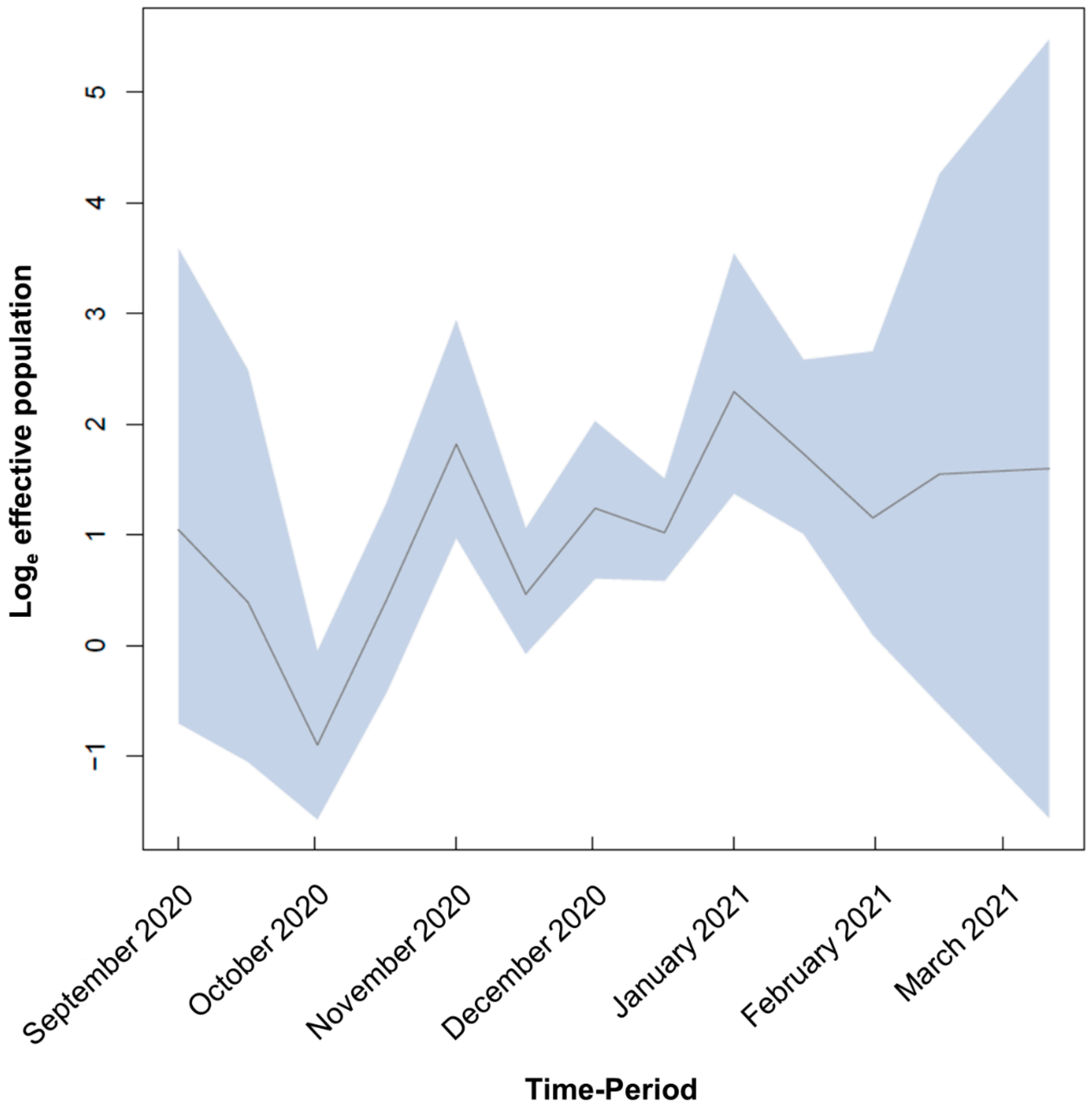

Figure S1. Population size changes in the large Cyprus-specific B.1.258 transmission lineage. The solid black line represents the estimated mean of the effective population size on a $\log_e$ scale (represented by the *y*-axis) through the sampling period from September 2020 to March 2021 (represented by the *x*-axis). The effective population size can be thought of as the number of individuals who contribute offspring to the descending generation. The blue-shaded area marks the associated 95% HPD interval.

Alpha
(B.1.1.7 & Q. sublineages)

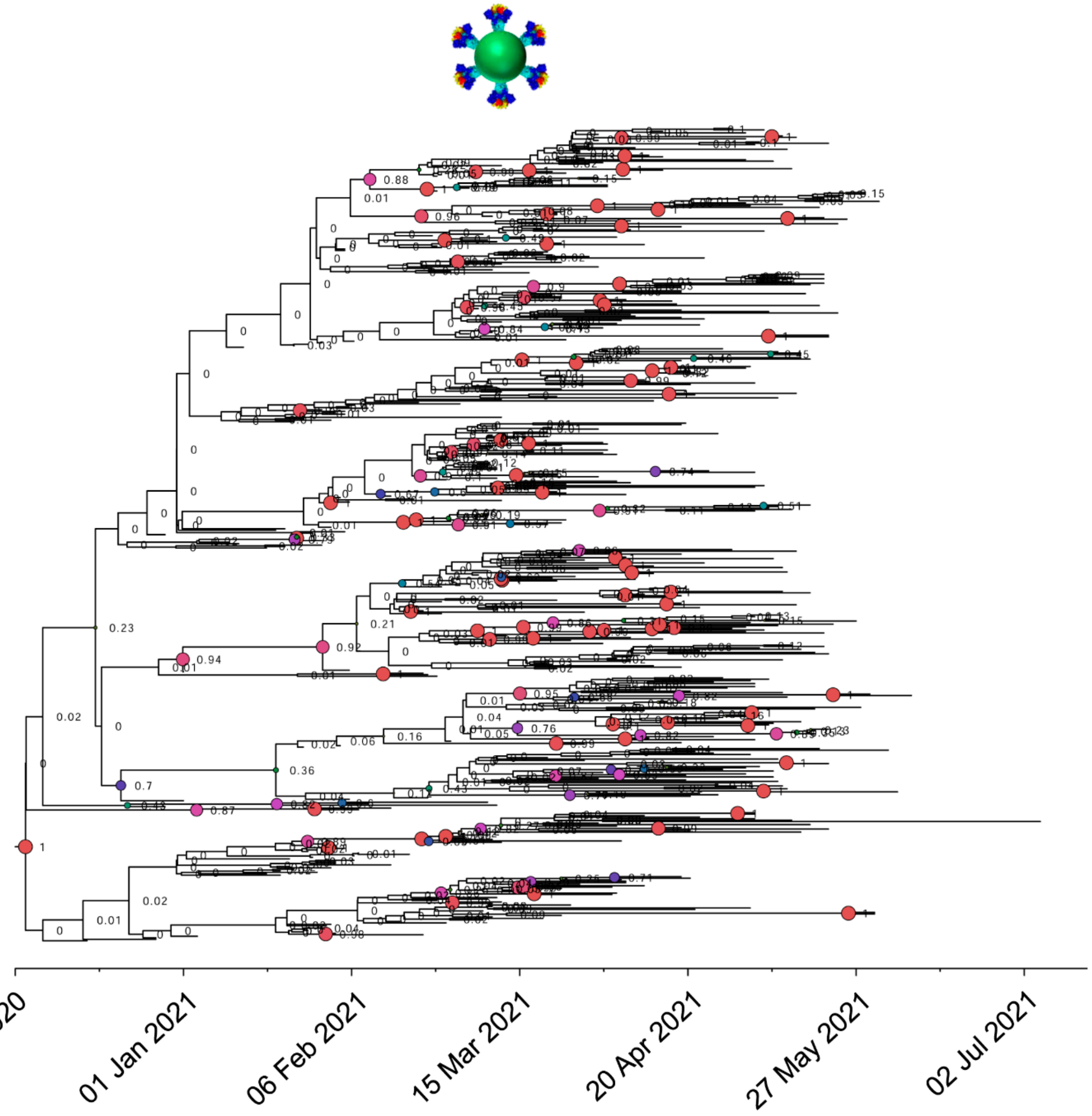

Figure S2. Maximum clade credibility summary tree of the large Cypriot-specific clade in the ML tree estimated form the Alpha (B.1.1.7 & Q. sublineages) data set. Several of the internal nodes that define large subclades are well-supported (red circles indicate high support of 1).

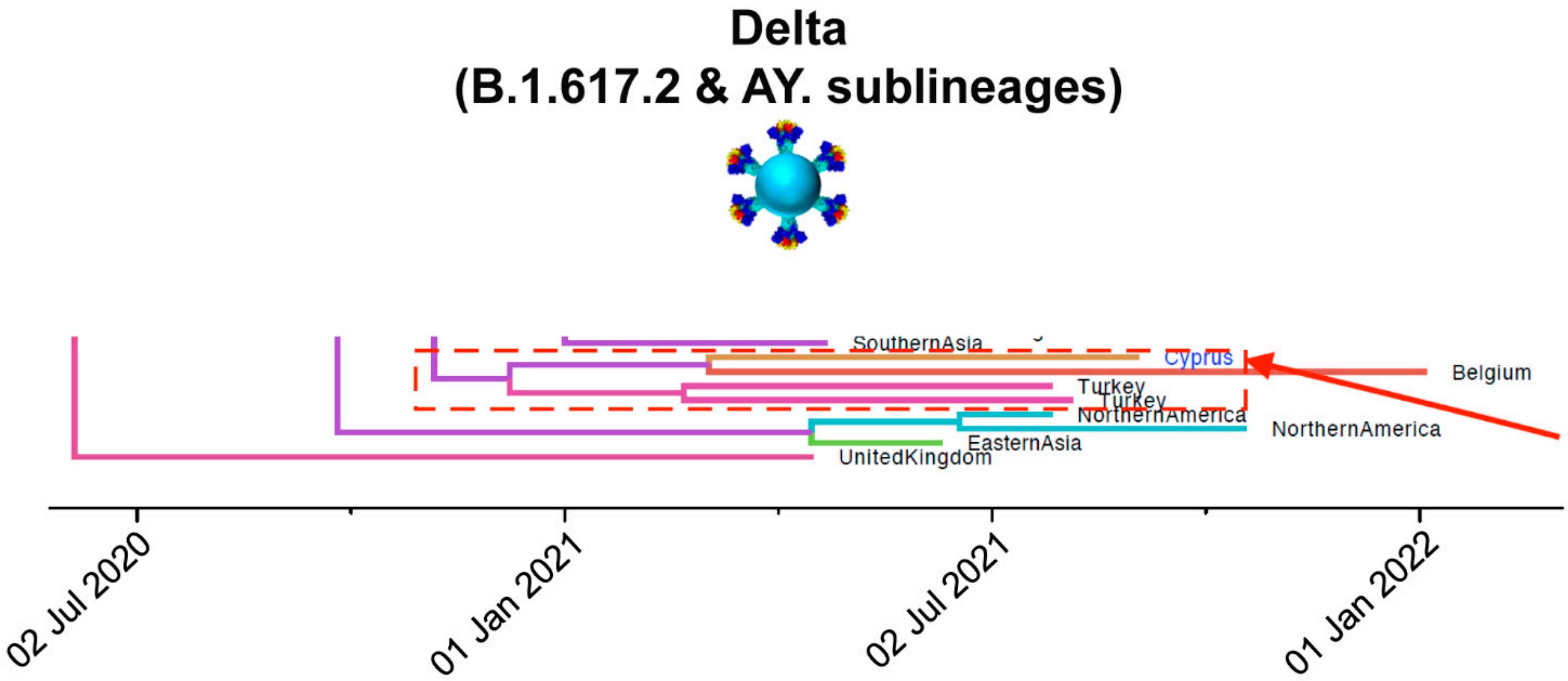

Figure S3. Inset of the Delta (B.1.617.2 & AY. sublineages) MCC summary tree. Branches in the dashed box are long and mostly represent several months of evolution. The red arrow indicates the Cypriot taxon that underlies the early estimate of the first introduction date of Delta into Cyprus. This figure is supplementary to Figure 6 (C) that shows the time-scaled migration history for the dataset of Delta (B.1.617.2 & AY. sublineages**).**

References

.1. Chen, C.; Nadeau, S.; Yared, M.; Voinov, P.; Xie, N.; Roemer, C.; Stadler, T. CoV-Spectrum: analysis of globally shared SARS-CoV-2 data to identify and characterize new variants. *Bioinformatics* **2022**, *38*, 1735–1737, doi:10.1093/bioinformatics/btab856.

2. Julia L. Mullen, Ginger Tsueng, Alaa Abdel Latif, Manar Alkuzweny, Marco Cano, Emily Haag, Jerry Zhou, Mark Zeller, Emory Hufbauer, Nate Matteson, Kristian G. Andersen, Chunlei Wu, Andrew I. Su, Karthik Gangavarapu, Laura D. Hughes, and the C. for V.S.B. outbreak.info Available online: https://outbreak.info/ (accessed on May 30, 2022).

3. Hodcroft, E.B. CoVariants: SARS-CoV-2 Mutations and Variants of Interest Available online: https://covariants.org/ (accessed on May 30, 2022).

4. Suratekar, R.; Ghosh, P.; Niesen, M.J.M.; Donadio, G.; Anand, P.; Soundararajan, V.; Venkatakrishnan, A.J. High diversity in Delta variant across countries revealed by genome-wide analysis of SARS-CoV-2 beyond the Spike protein. *Mol. Syst. Biol.* **2022**, doi:10.15252/msb.202110673.
